# Retrotransposons are co-opted to activate hematopoietic stem cells and erythropoiesis

**DOI:** 10.1126/science.ado6836

**Published:** 2024-11-08

**Authors:** Julia Phan, Brandon Chen, Zhiyu Zhao, Gabriele Allies, Antonella Iannaccone, Animesh Paul, Feyza Cansiz, Alberto Spina, Anna-Sophia Leven, Alexandra Gellhaus, Dirk Schadendorf, Rainer Kimmig, Marcel Mettlen, Alpaslan Tasdogan, Sean J. Morrison

**Affiliations:** 1 Children’s Research Institute and the Department of Pediatrics, University of Texas Southwestern Medical Center; Dallas, TX 75390, USA.; 2 Department of Dermatology, University Hospital Essen & German Cancer Consortium; Essen, & National Center for Tumor Diseases (NCT-West), Campus Essen & Research Alliance Ruhr, Research Center One Health, University Duisburg-Essen, Campus Essen, Essen, Germany.; 3 Department of Gynecology and Obstetrics, University Hospital Essen, 45147 Essen, Germany.; 4 Department of Cell Biology, University of Texas Southwestern Medical Center; Dallas, Texas 75235-9039.; 5 Howard Hughes Medical Institute, University of Texas Southwestern Medical Center; Dallas, TX 75390, USA.

## Abstract

Hematopoietic stem cells (HSCs) and erythropoiesis are activated during pregnancy and after bleeding by the derepression of retrotransposons, including endogenous retroviruses and LINE elements. Retrotransposon transcription activates the innate immune sensors cGAS and STING, which induce interferon and interferon regulated genes in HSCs, increasing HSC division and erythropoiesis. Inhibition of reverse transcriptase or deficiency for cGAS or STING had little or no effect on hematopoiesis in non-pregnant mice but depleted HSCs and erythroid progenitors in pregnant mice, reducing red blood cell counts. Retrotransposons and interferon regulated genes were also induced in mouse HSCs after serial bleeding and in human HSCs during pregnancy. Reverse transcriptase inhibitor use was associated with anemia in pregnant, but not non-pregnant, people suggesting conservation of these mechanisms from mice to humans.

## One sentence summary

Retrotransposon transcription increases in hematopoietic stem cells during erythropoietic stresses, activating cGAS-STING signaling, increasing cell division and erythropoiesis, and preventing anemia.

## Graphical Abstract

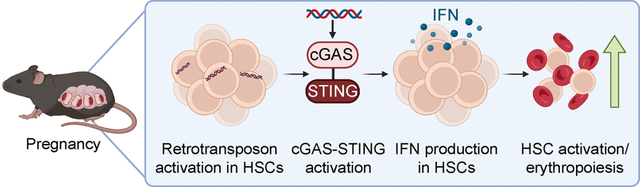

## Introduction

Hematopoietic stem cells (HSCs) are activated by hematopoietic stresses to increase blood cell production, though there is a limited understanding of the underlying mechanisms. For example, during pregnancy, estrogen/estrogen receptor signaling activates HSCs, increasing HSC cell division and inducing extramedullary erythropoiesis in the spleen (1). This is necessary to maintain red blood cell counts as estrogen receptor deficiency in hematopoietic cells leads to maternal anemia. Serial bleeding also increases erythropoiesis, partly by increasing erythropoietin production (2, 3), though erythropoietin is not sufficient to explain the HSC activation or extramedullary hematopoiesis observed after substantial blood loss (4).

During embryonic development, sterile inflammation caused by transposable element transcription and interferon expression, promotes the formation of HSCs (5–9). Transposable elements, including DNA transposons and retrotransposons, are germline DNA elements that are capable of moving from one location to another in the genome (10). Retrotransposons, including endogenous retroviruses, long interspersed nuclear elements (LINEs) and short interspersed nuclear elements (SINEs), encode reverse transcriptase, which reverse transcribes retrotransposon transcripts into DNA, enabling reinsertion into the genome. The RNA and/or reverse-transcribed DNA that are produced as a result of transposable element transcription can activate innate immune sensors, including Toll-like receptors (11, 12), retinoic acid inducible gene I (RIG-I) (13–15), melanoma differentiation-associated protein 5 (MDA5) (16–18), cyclic GMP-AMP synthase (cGAS) and stimulator of interferon genes (STING) (19–21). In turn, these innate immune sensors promote the expression of interferon and other proinflammatory cytokines (22).

In adult hematopoietic cells, aging (23, 24), irradiation (13, 25), chemotherapy (17), and drugs that inhibit DNA methylation (18, 26) or topoisomerase (27) lead to DNA damage, changes in chromatin structure, and increased expression of transposable elements. Retrotransposon expression in hematopoietic cells activates inflammatory pathways as a result of signaling by MDA5 (17) and RIG-I (14), promoting HSC activation after chemotherapy (17) or bacterial infection (28), though this may undermine long-term HSC function by depleting HSC self-renewal potential (29–31). Deficiency for MDA5, cGAS, or STING has little effect on HSC function or hematopoiesis under normal conditions (17, 28, 32) raising the question of whether these pathways regulate HSC function or hematopoiesis in normal adult mammals and whether transposons contribute to the regeneration of adult hematopoiesis.

## Results

### Pregnancy induced retrotransposon transcription in HSCs in mice

**Fig. 1.**
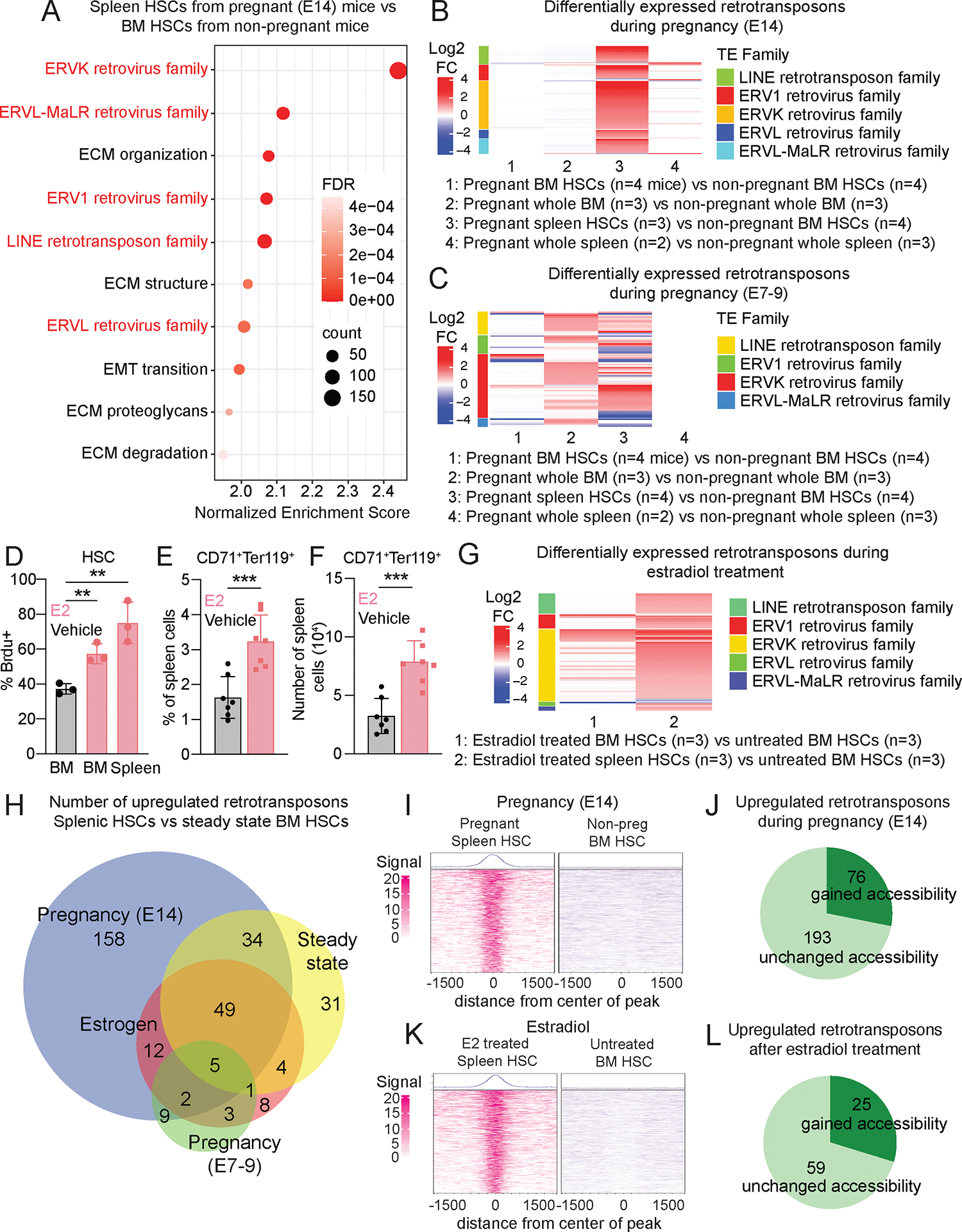
Retrotransposon transcription increases in HSCs during pregnancy. **(A, B)** We performed RNA sequencing in bone marrow HSCs and unfractionated bone marrow and spleen cells from pregnant and non-pregnant female mice as well as spleen HSCs from pregnant mice (spleen HSCs are very rare in non-pregnant mice). (**A**) Five out of the 7 most highly enriched gene sets in spleen HSCs from pregnant dams (E14) as compared to bone marrow HSCs from non-pregnant mice were endogenous retrotransposon sequences from multiple families (NES > 2 and FDR <0.01). **(B)** The list of retrotransposons that differed (log2 fold change > 1, FDR < 0.05) among the cell populations in the analysis showed that retrotransposons were broadly de-repressed in splenic HSCs from pregnant mice. (**C**) We performed a similar analysis in pregnant dams at an earlier stage of pregnancy (E7–9) and again found a broad de-repression (log2 fold change > 1, FDR< 0.05) of multiple families of retrotransposons in splenic HSCs from pregnant dams. Some of these retrotransposons were also more highly expressed in bone marrow HSCs from pregnant as compared to non-pregnant mice. **(D)** The percentage of HSCs that incorporated a 72 hour pulse of BrdU in estradiol-treated or vehicle control mice (3 mice per treatment). **(E, F)** The frequencies (**E**) and numbers (**F**) of CD71^+^Ter119^+^ erythroid progenitors in the spleens of estradiol-treated or control mice (a total of 7 mice per treatment from three independent experiments). The flow cytometry gates are shown in Figs. S1 and S2. In panels D-F, each dot represents a different mouse and data represent mean ± standard deviation (*p < 0.05; **p < 0.01; ***p < 0.001). **(G)** Retrotransposons that changed (log2 fold change > 1, FDR< 0.05) in expression in bone marrow or spleen HSCs from estradiol-treated as compared to untreated control mice. **(H)** Venn diagram showing the overlap of retrotransposons that were increased in expression (log2 fold change > 1, FDR <0.05) in splenic HSCs from pregnant (E14) mice, normal non-pregnant mice (steady state), early (E7–9) pregnant mice, and estradiol-treated mice all compared to bone marrow HSCs from normal, non-pregnant mice. **(I-J)** ATAC-sequencing of spleen HSCs from pregnant mice as compared to bone marrow HSCs from non-pregnant mice showing differentially accessible regions (**I**) and the number of upregulated retrotransposons in spleen HSCs from pregnant mice that overlapped with genomic regions that gained accessibility (**J**). **(K-L)** ATAC-sequencing of spleen HSCs from estradiol-treated mice as compared to bone marrow HSCs from untreated mice showing differentially accessible regions (**K**) and the number of upregulated retrotransposons in spleen HSCs from estradiol-treated mice that overlapped with genomic regions that gained accessibility (**L**). The numbers of mice from which cells were isolated for RNA sequencing is shown in panels **B**, **C**, and **G**. To isolate spleen HSCs from normal non-pregnant mice, spleens were pooled from 7–12 mice per replicate for a total of 3 replicates **(H)**. The statistical significance of differences among treatments was assessed using Student’s *t*-tests with Holm-Sidak’s multiple comparisons adjustments **(D)** and Student’s *t*-tests (**E, F)**. All statistical tests were two-sided.

To identify transcriptional changes that occur within HSCs during pregnancy, we performed RNA sequencing on CD150^+^CD48^−^Lineage^−^Sca-1^+^c-kit^+^ HSCs and unfractionated cells from the bone marrow and spleens of pregnant dams at embryonic day (E)14 of pregnancy as well as from non-pregnant female control mice. We assessed both coding sequences and transposable elements (33). In splenic HSCs from pregnant mice as compared to bone marrow HSCs from non-pregnant mice, 5 out of the 7 most highly upregulated gene sets were retrotransposons, including endogenous retroviruses (ERVK, ERVL-MaLR, ERV1, and ERVL families) and LINE elements (Fig. 1A). These retrotransposons were transcribed mainly in splenic HSCs, not in bone marrow HSCs, whole bone marrow cells, or whole spleen cells (Fig. 1B, Table S1). At earlier stages of pregnancy (E7-E9) we observed similar results, with increased retrotransposon expression in splenic HSCs from pregnant mice, and to a lesser extent in bone marrow HSCs and whole bone marrow cells from pregnant mice (Fig. 1C).

We also performed RNA-sequencing in splenic HSCs from pregnant mice versus splenic HSCs from non-pregnant mice, along with bone marrow HSCs and unfractionated bone marrow and spleen cells from pregnant and non-pregnant mice (Fig. S3A). Gene sets for the LINE, ERVK, and ERVL-MaLR retrotransposon families were enriched in splenic HSCs from pregnant mice as compared to splenic HSCs from non-pregnant mice (Fig. S3B), but not in bone marrow HSCs, whole bone marrow, or whole spleen cells from pregnant versus non-pregnant mice. Spleen HSCs from pregnant mice, thus, exhibited increased retrotransposon expression as compared to spleen HSCs from non-pregnant mice.

To address whether retrotransposons were derepressed in other hematopoietic progenitors besides HSCs, we performed RNA-sequencing on megakaryocyte-erythroid progenitors (MEPs), CD71^+^Ter119^+^ erythroid progenitors, and unfractionated cells from the bone marrow and spleen of pregnant dams (Fig. S3C). Retrotransposon expression did not increase during pregnancy in CD71^+^Ter119^+^ cells or unfractionated cells from the bone marrow or spleen (Fig. S3A–C). However, we did observe increased LINE expression in bone marrow MEPs from pregnant as compared to non-pregnant mice (Fig. S3D). This suggests that retrotransposons were derepressed mainly in HSCs during pregnancy and to a lesser extent in some hematopoietic progenitors.

To test if estrogen contributed to the derepression of retrotransposons during pregnancy, we treated mice with estradiol or vehicle. Consistent with our prior study (1), estradiol (2ug/day for 6 days) administration increased cell division by HSCs in the bone marrow and spleen (Fig. 1D) as well as erythropoiesis in the spleen (Fig. 1E–F). We performed RNA sequencing on HSCs and unfractionated cells from the bone marrow and spleen of estradiol-treated (2ug/day for 13 days) versus untreated control mice. Retrotransposon expression increased in splenic HSCs, and to a lesser extent in bone marrow HSCs, from estradiol-treated as compared to untreated mice (Fig. 1G). Most of the retrotransposons that were upregulated in HSCs during early (E7–9) pregnancy and nearly all of the retrotransposons that were upregulated in response to estradiol treatment were also upregulated during late (E14) pregnancy (Figure 1H). This suggested that estrogen signaling was part of the mechanism by which retrotransposons are derepressed during pregnancy; however, the number of retrotransposons that were expressed after estradiol treatment was much smaller than during pregnancy (Fig. 1H), suggesting other mechanisms also contribute.

### Serial bleeding of mice also derepressed retrotransposons in HSCs

To test if other erythropoietic stresses also induced retrotransposon expression in HSCs we performed RNA-sequencing on HSCs and unfractionated cells from the bone marrow and spleen of serially-bled and non-bled control mice (Fig. S4). Gene sets related to erythropoiesis were enriched in splenic HSCs from serially-bled mice as compared to bone marrow and spleen HSCs from control mice (e.g. Fig. S4A). Similar to what we observed during pregnancy, 265 retrotransposons were increased in expression in splenic HSCs from serially-bled mice as compared to bone marrow HSCs from non-bled mice (Fig. S4B and S4C). Few retrotransposons were increased in expression in bone marrow HSCs from serially-bled mice (Fig. S4B and S4C). Retrotransposons were expressed in spleen HSCs from serially-bled mice at levels that were nearly as high as during pregnancy (Figs. 1B, S4C and S4D) and were mainly from the same retrotransposon families (Fig. S4D and S4E). Our data thus indicated that retrotransposons were derepressed in HSCs after erythropoietic stresses.

### The accessibility of loci that encode retrotransposons increased in HSCs during pregnancy

To begin to understand how retrotransposon expression increased during pregnancy, we performed ATAC-sequencing on HSCs from the bone marrow and spleen of pregnant versus non-pregnant mice (Fig. 1I). We observed little or no change in chromatin accessibility in bone marrow HSCs from pregnant as compared to non-pregnant mice. Conversely, approximately 1100 genomic regions (0.07% of the genome) were more accessible in spleen HSCs from pregnant mice as compared to bone marrow HSCs from non-pregnant mice (Fig. 1I). We used bone marrow HSCs from non-pregnant mice as a comparison as HSCs were so rare in the spleens of non-pregnant mice that we did not obtain interpretable ATAC-seq profiles. These regions with increased accessibility overlapped with 28% of the retrotransposons that were upregulated in spleen HSCs from pregnant mice (Fig. 1J). The remaining 72% of upregulated retrotransposons overlapped with regions that showed a trend toward increased accessibility but the differences were not statistically significant. No regions became less accessible in spleen HSCs from pregnant mice as compared to bone marrow HSCs from non-pregnant mice. Loci that encoded retrotransposons, thus, preferentially became more accessible during pregnancy.

We also performed ATAC-sequencing comparing spleen HSCs from estradiol-treated mice to bone marrow HSCs from untreated control mice (Fig. 1K). Similar to during pregnancy, there was little or no change in chromatin accessibility in bone marrow HSCs from estradiol-treated as compared to control mice. Approximately 2100 genomic regions (0.22% of the genome) became more accessible in spleen HSCs from estradiol-treated mice as compared to bone marrow HSCs from control mice (Fig. 1K; no regions became less accessible). These regions overlapped with 30% of the retrotransposons that were upregulated in spleen HSCs from estradiol-treated mice (Fig. 1L). The remaining 70% of retrotransposons overlapped with regions that showed a trend towards increased accessibility but the differences were not statistically significant. Estradiol treatment thus appeared to preferentially increase the accessibility of loci that contain retrotransposons. ERα binding is known to remodel chromatin and increase the accessibility of specific genomic regions (34) and chromatin structure regulates the transcription of endogenous retroviruses (35). Nonetheless, estradiol may influence the expression of retrotransposons through additional mechanisms.

### Antiretroviral treatment reduced HSCs and erythropoiesis during pregnancy

**Fig. 2:**
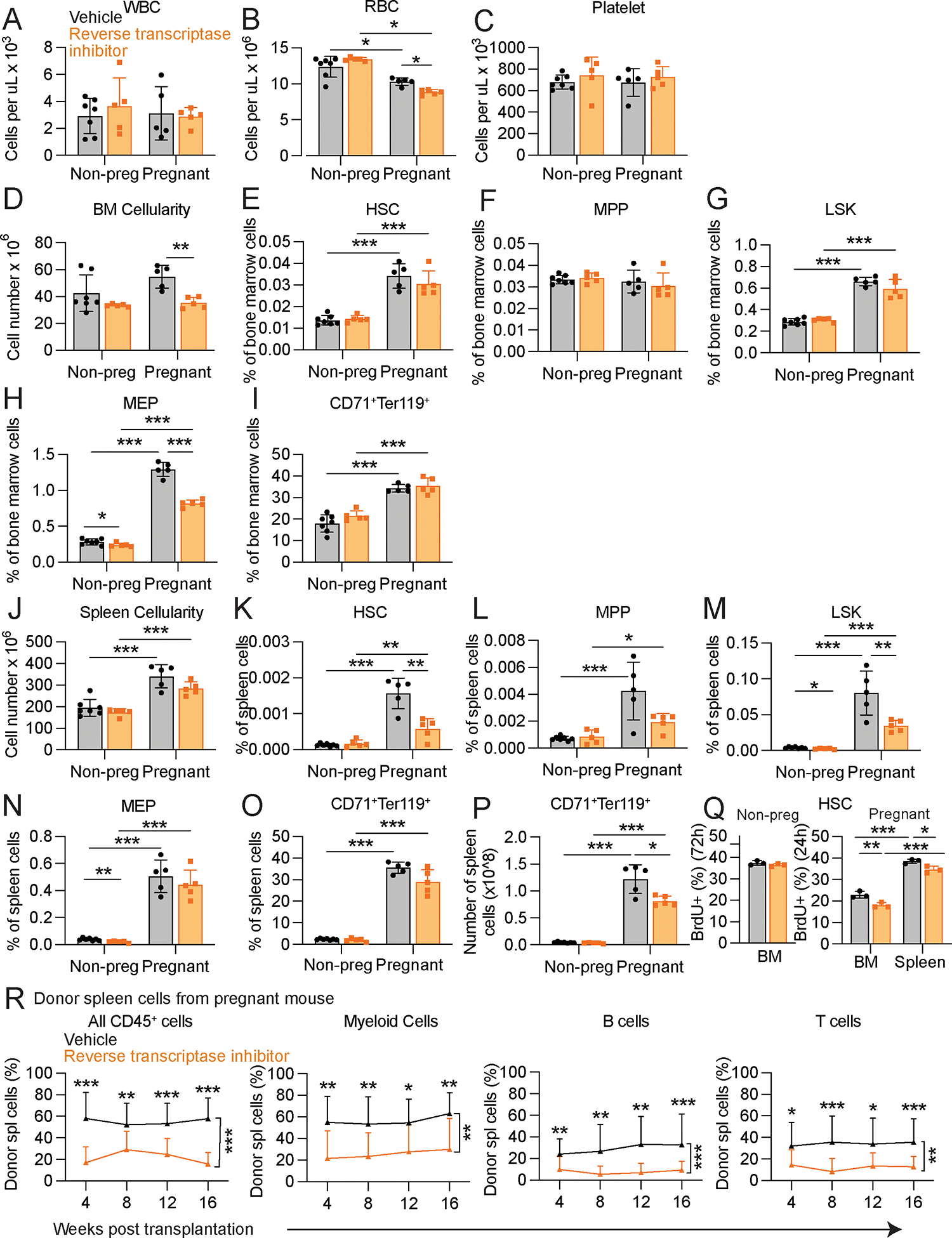
Reverse transcriptase inhibitors had no effect on hematopoiesis in non-pregnant mice but decreased splenic HSCs and erythropoiesis in pregnant mice, leading to anemia. Pregnant and non-pregnant female mice were treated with reverse transcriptase inhibitors versus vehicle control (panels **A – P** reflect n=5 to 7 mice per treatment in 2 independent experiments; each dot represents a different mouse): (**A-C**) blood cell counts, (**D-I**) bone marrow cellularity in one tibia and one femur (**D**) and the frequencies of HSCs (**E**), MPPs (**F**), LSK cells (**G**), MEPs (**H**), and CD71^+^Ter119^+^ erythroid progenitors (**I**) in the bone marrow. (**J-O**) Spleen cellularity (**J**) and the frequencies of HSCs (**K**), MPPs (**L**), LSK cells (**M**), MEPs (**N**), and CD71^+^Ter119^+^ erythroid progenitors (**O**) in the spleen. **(P)** Number of CD71^+^Ter119^+^ erythroid progenitors in the spleen. **(Q)** The percentage of HSCs that incorporated a 72 hour (non-pregnant) or 24 hour (pregnant) pulse of BrdU in reverse transcriptase inhibitor or vehicle-treated mice (3 mice per treatment). (**R**) Donor cell reconstitution of CD45^+^ hematopoietic cells, Mac-1^+^Gr-1^+^ myeloid cells, B220^+^ B cells, and CD3^+^ T cells in the blood of mice that were competitively transplanted with 1.5 × 10^6^ donor spleen cells from pregnant dams that were treated with reverse transcriptase inhibitors or vehicle control (3 donor mice and a total of 13–14 recipients per treatment in 3 independent experiments). The flow cytometry gates are shown in Figs. S1 and S2. All data represent mean ± standard deviation (*p < 0.05; **p < 0.01; ***p < 0.001). Statistical significance was assessed using two-way ANOVAs followed by Sidak’s multiple comparisons adjustments **(A, C, E-O),** Mann-Whitney tests followed by Holm-Sidak’s multiple comparisons adjustment **(B),** Welch’s *t*-tests followed by Holm-Sidak’s multiple comparisons adjustments **(D, P)**, a Student’s *t*-test **(Q:** non-pregnant) and a matched samples two-way ANOVA followed by Sidak’s multiple comparisons adjustment **(Q:** pregnant**)**, or nparLD tests followed by Holm-Sidak’s multiple comparisons adjustments for overall differences taking into account all time points and Mann-Whitney tests for data at individual time points **(R)**. All statistical tests were two-sided.

Retrotransposon transcription can lead to the accumulation of DNA:RNA hybrids and double stranded DNAs in the cytoplasm as a result of reverse transcription (20, 36). To test if reverse transcription contributed to HSC activation and erythropoiesis, we treated non-pregnant and pregnant mice with tenofovir and emtricitabine, reverse transcriptase inhibitors that are used clinically to suppress human immunodeficiency virus (HIV) replication (37). These inhibitors also have activity against reverse transcriptases in mouse and human retrotransposons (38). In non-pregnant female mice, reverse transcriptase inhibitors had little or no effect on blood cell counts (Fig. 2A–C), or bone marrow (Fig. 2D–I) or spleen (Fig. 2J–O) hematopoiesis, including overall cellularity and the frequencies of HSCs, multipotent progenitors (MPPs), Lineage^−^Sca-1^+^c-kit^+^ (LSK) progenitors, MEPs, and CD71^+^Ter119^+^ erythroid progenitors in the bone marrow and spleen. In contrast, treatment of pregnant dams (E9–14) with reverse transcriptase inhibitors reduced red blood cell counts without affecting white blood cell or platelet counts (Fig. 2A–C). Treatment with reverse transcriptase inhibitors also reduced bone marrow cellularity and the frequency of MEPs in the bone marrow (Fig. 2D–I) as well as the frequencies of HSCs and LSK cells in the spleen (Fig. 2J–O). The absolute number of CD71^+^Ter119^+^ erythroid progenitors declined in the spleens of pregnant mice treated with reverse transcriptase inhibitors (Fig. 2P) as did the rates of HSC cell division in the bone marrow and spleen of pregnant mice (Fig. 2Q).

Consistent with the decrease in HSC frequency in the spleen (Fig. 2K), but not the bone marrow (Fig. 2E), treatment of pregnant mice with reverse transcriptase inhibitors reduced the reconstituting activity of spleen cells (Fig. 2R), but not bone marrow cells (Fig. S5A), upon competitive transplantation into irradiated mice. Treatment of non-pregnant mice with reverse transcriptase inhibitors had no effect on the reconstituting potential of bone marrow (Fig. S5B) or spleen (Fig. S5C) cells. Reverse transcription was, thus, necessary to increase HSC cell division and splenic erythropoiesis and to maintain normal red blood cell counts during pregnancy.

### STING signaling promoted HSC proliferation and erythropoiesis during pregnancy

To test if retrotransposons activated cGAS-STING signaling in HSCs, we performed immunofluorescence analysis using anti-phosphoSTING antibody on HSCs that were flow cytometrically isolated from the bone marrow and spleen of pregnant and non-pregnant female mice. STING is phosphorylated when activated (39). We observed punctate phosphoSTING staining in the cytoplasm of HSCs from the bone marrow and spleen of pregnant mice at levels that were higher than in HSCs from non-pregnant mice or *STING*^*gt/gt*^ mice (Fig. S6A and S6B). *STING*^*gt/gt*^ mice have a missense mutation in exon 6 which eliminates STING protein (40). Treatment of pregnant mice with reverse transcriptase inhibitors reduced phosphoSTING staining in HSCs from the spleen but not the bone marrow (Fig. S6C and S6D). STING was thus activated in HSCs from the bone marrow and spleen of pregnant mice and reverse transcriptase inhibitors reduced STING activation in the spleen.

**Fig. 3:**
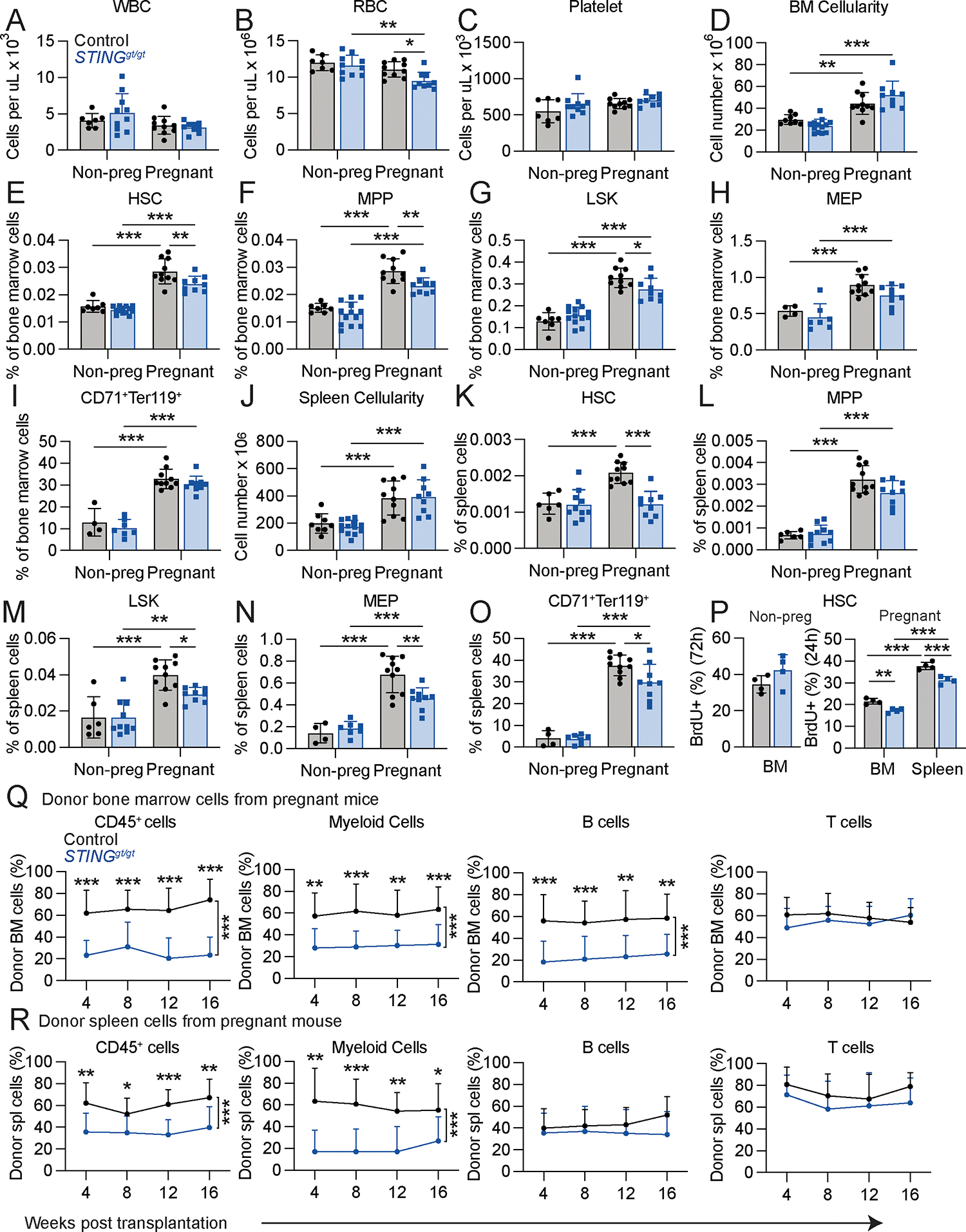
STING is necessary to increase HSC frequency and erythropoiesis during pregnancy. We assessed hematopoiesis in pregnant or non-pregnant female *STING*^*gt/gt*^ or littermate control mice (all panels reflect 4 to 13 mice per treatment in 4 independent experiments; each dot represents a different mouse): (**A**-**C**) blood cell counts, (**D-I**) bone marrow cellularity in one tibia and one femur (**D**) and the frequencies of HSCs (**E**), MPPs (**F**), LSK cells (**G**), MEPs (**H**), and CD71^+^Ter119^+^ erythroid progenitors (**I**) in the bone marrow. (**J-O**) Spleen cellularity (**J**) and the frequencies of HSCs (**K**), MPPs (**L**), LSK cells (**M**), MEPs (**N**), and CD71^+^Ter119^+^ erythroid progenitors (**O**) in the spleen. (**P**) The percentage of HSCs that incorporated a 72 hour (non-pregnant) or 24 hour (pregnant) pulse of BrdU in *STING*^*gt/gt*^ and littermate control mice (4 mice per treatment in 2 independent experiments). (**Q**) Donor cell reconstitution of CD45^+^ hematopoietic cells, Mac-1^+^Gr-1^+^ myeloid cells, B220^+^ B cells, and CD3^+^ T cells in the blood of mice that were competitively transplanted with 5 × 10^5^ donor bone marrow cells from pregnant *STING*^*gt/gt*^ or littermate control dams (3 donor mice were transplanted into a total of 13–14 recipients per genotype in 3 independent experiments). (**R**) Donor cell reconstitution in the blood of mice that were competitively transplanted with 1.5 × 10^6^ donor spleen cells from pregnant *STING*^*gt/gt*^ or littermate control dams (3 donor mice were transplanted into a total of 11–12 recipients per genotype in 3 independent experiments). The flow cytometry gates are shown in Figs. S1 and S2. All data represent mean ± standard deviation (*p < 0.05; **p < 0.01; ***p < 0.001).. Statistical significance was assessed two-way ANOVAs followed by Sidak’s multiple comparisons adjustments (**A-B, D-K, M**), Welch’s *t*-tests followed by Holm-Sidak’s multiple comparisons adjustments (**C, L, O**), multiple Student’s *t*-tests followed by Holm-Sidak’s multiple comparisons adjustments (**N**), a Student’s *t*-test **(P:** non-pregnant) and a matched samples two-way ANOVA followed by Sidak’s multiple comparisons adjustment **(P:** pregnant**),** or nparLD tests followed by Holm-Sidak’s multiple comparisons adjustments for overall differences taking into account all time points and Mann-Whitney tests for data at individual time points **(Q, R).** All statistical tests were two-sided.

To test if STING was necessary for increased erythropoiesis during pregnancy, we examined *STING*^*gt/gt*^ and littermate control mice. In non-pregnant female mice, STING deficiency had no effect on blood cell counts (Fig. 3A–C), bone marrow (Fig. 3D) or spleen (Fig. 3J) cellularity, or the frequencies of HSCs, MPPs, LSK cells, MEPs, or CD71^+^Ter119^+^ erythroid progenitors in the bone marrow (Fig. 3E–I) or spleen (Fig. 3K–O). In non-pregnant mice, STING deficiency also had no effect on the rate of HSC cell division (Fig. 3P) or on the reconstituting potential of bone marrow (Fig. S7A) or spleen (Fig. S7B) cells upon competitive transplantation into irradiated mice. In pregnant mice, STING deficiency reduced red blood cell counts but not white blood cell or platelet counts (Fig. 3A–C). In pregnant mice, STING deficiency did not reduce bone marrow (Fig. 3D) or spleen (Fig. 3J) cellularity and it had a small, but statistically significant, effect on the frequencies of HSCs and restricted hematopoietic progenitors in the bone marrow (Fig. 3E–I). STING deficiency reduced the frequencies of HSCs, LSK cells, MEPs, and CD71^+^Ter119^+^ erythroid progenitors in the spleens of pregnant mice to a greater extent (Fig. 3K–O). STING deficiency also reduced the rates of HSC cell division in the bone marrow and spleen of pregnant mice (Fig. 3P). The reconstituting potential of STING deficient bone marrow (Fig. 3Q) and spleen (Fig. 3R) cells from pregnant mice was reduced as compared to control cells from pregnant mice upon competitive transplantation into irradiated mice. STING activation, thus, contributed to the increased HSC proliferation and erythropoiesis in the spleen during pregnancy such that STING deficiency led to lower red blood cell counts in pregnant mice.

### cGAS signaling promoted HSC proliferation and erythropoiesis during pregnancy

**Fig. 4:**
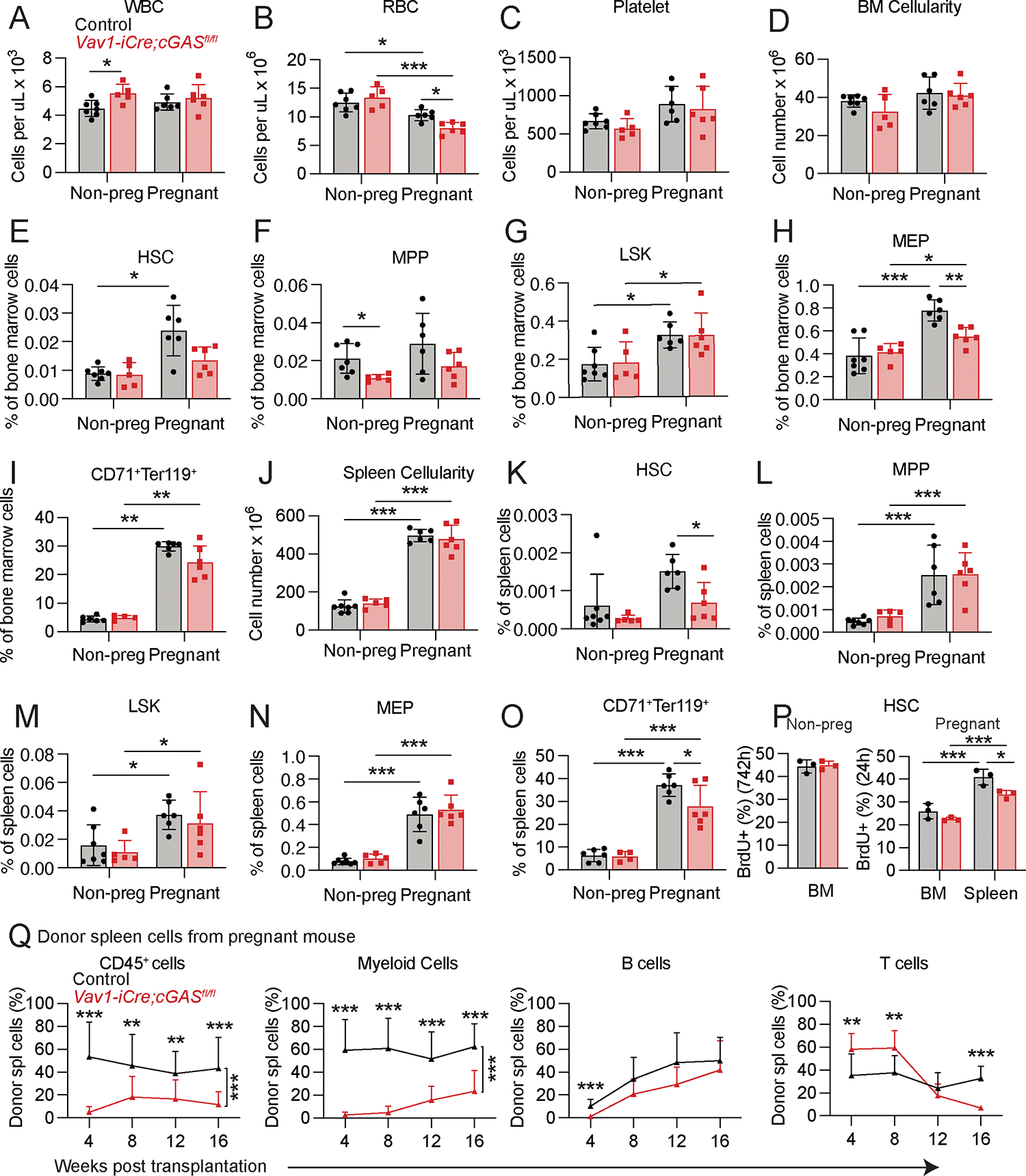
cGAS is necessary to increase HSC frequency and erythropoiesis during pregnancy. We assessed hematopoiesis in pregnant or non-pregnant female *Vav1-iCre;cGAS*^*fl/fl*^ or littermate control mice (all panels reflect 4 to 7 mice per treatment in 3 independent experiments; each dot represents a different mouse): (**A-C**) blood cell counts, (**D-I**) bone marrow cellularity in one tibia and one femur (**D**) and the frequencies of HSCs (**E**), MPPs (**F**), LSK cells (**G**), MEPs (**H**), and CD71^+^Ter119^+^ erythroid progenitors (**I**) in the bone marrow. (**J-O**) Spleen cellularity (**J**) and the frequencies of HSCs (**K**), MPPs (**L**), LSK cells (**M**), MEPs (**N**), and CD71^+^Ter119^+^ erythroid progenitors (**O**) in the spleen. (**P**) The percentage of HSCs that incorporated a 72 hour (non-pregnant) or 24 hour (pregnant) pulse of BrdU in *Vav1-iCre;cGAS*^*fl/fl*^ or littermate control mice (3 mice per treatment). **(Q)** Donor cell reconstitution of CD45^+^ hematopoietic cells, Mac-1^+^Gr-1^+^ myeloid cells, B220^+^ B cells, and CD3^+^ T cells in the blood of mice that were competitively transplanted with 1.5 × 10^6^ donor spleen cells from pregnant *Vav1-iCre;cGAS*^*fl/fl*^ or littermate control dams (3 donor mice were transplanted into a total of 12–13 recipients per genotype in 3 independent experiments). The flow cytometry gates are shown in Figs. S1 and S2. All data represent mean ± standard deviation (*p < 0.05; **p < 0.01; ***p < 0.001). Statistical significance was assessed using two-way ANOVAs followed by Sidak’s multiple comparisons adjustments (**A-D, J, L-O),** Mann-Whitney tests followed by Holm-Sidak’s multiple comparisons adjustments **(E, I, K),** multiple Student’s *t*-tests followed by Holm-Sidak’s multiple comparisons adjustments **(F-H, K),** a Student’s *t*-test **(P:** non-pregnant) and a matched samples two-way ANOVA followed by Sidak’s multiple comparisons adjustment **(P:** pregnant**)**, or nparLD tests followed by Holm-Sidak’s multiple comparisons adjustments for overall differences taking into account all time points and Mann-Whitney tests for data at individual time points (**Q).** All statistical tests were two-sided.

In non-pregnant female mice, cGAS deficiency had little or no effect on blood cell counts (Fig. 4A–C), bone marrow (Fig. 4D) or spleen (Fig. 4J) cellularity, or the frequencies of HSCs, MPPs, LSK cells, MEPs, or CD71^+^Ter119^+^ erythroid progenitors in the bone marrow (Fig. 4E–I) or spleen (Fig. 4K–O). cGAS deficiency also had no effect on the rate of HSC cell division (Fig. 3P) or the reconstituting potential of bone marrow (Fig. S8B) or spleen (Fig. S8C) cells upon competitive transplantation into irradiated mice. Conversely, in pregnant mice, cGAS deficiency reduced red blood cell, but not white blood cell or platelet counts (Fig. 4A–C). In pregnant mice, cGAS deficiency did not reduce bone marrow (Fig. 4D) or spleen (Fig. 4J) cellularity but reduced the frequencies of MEPs in the bone marrow (Fig. 4E–I) as well as HSCs and CD71^+^Ter119^+^ erythroid progenitors in the spleen (Fig. 4K–O). cGAS deficiency also reduced the rate of HSC cell division in the spleens of pregnant mice (Fig. 4P). The reconstituting potential of cGAS deficient spleen (Fig. 4Q), but not bone marrow (Fig. S8A), cells from pregnant mice was reduced as compared to control cells from pregnant mice. cGAS activation, thus, contributed to increased HSC cell division and erythropoiesis in the spleen during pregnancy.

### cGAS-STING-signaling promoted interferon expression in HSCs from pregnant mice

cGAS-STING activation leads to the production of interferons (IFN) (19, 41, 42). An acute increase in IFNα production promotes HSC proliferation, while chronic IFNα production erodes HSC self-renewal potential and depletes HSCs (30, 43). To test if IFNs increased during pregnancy, we performed enzyme-linked immunosorbent assays (ELISAs) for IFNα in blood plasma, bone marrow lysate, and spleen lysate from female wild-type, *STING*^*gt/gt*^, and *Vav1-iCre;cGAS*^*fl/fl*^ mice that were pregnant or non-pregnant. Pregnancy increased IFNα levels in bone marrow and spleen lysate, but not blood plasma, from wild-type mice (Fig. S9A–F). These increases were not detected in *STING*^*gt/gt*^ or *Vav1-iCre;cGAS*^*fl/fl*^ mice (Fig. S9A–F). We did not detect increases in the levels of other inflammatory cytokines in blood plasma, bone marrow or spleen lysate from pregnant as compared to non-pregnant mice (Fig. S10). Therefore, there was a cGAS-STING dependent increase in IFNα levels during pregnancy, particularly in the spleen.

**Fig. 5:**
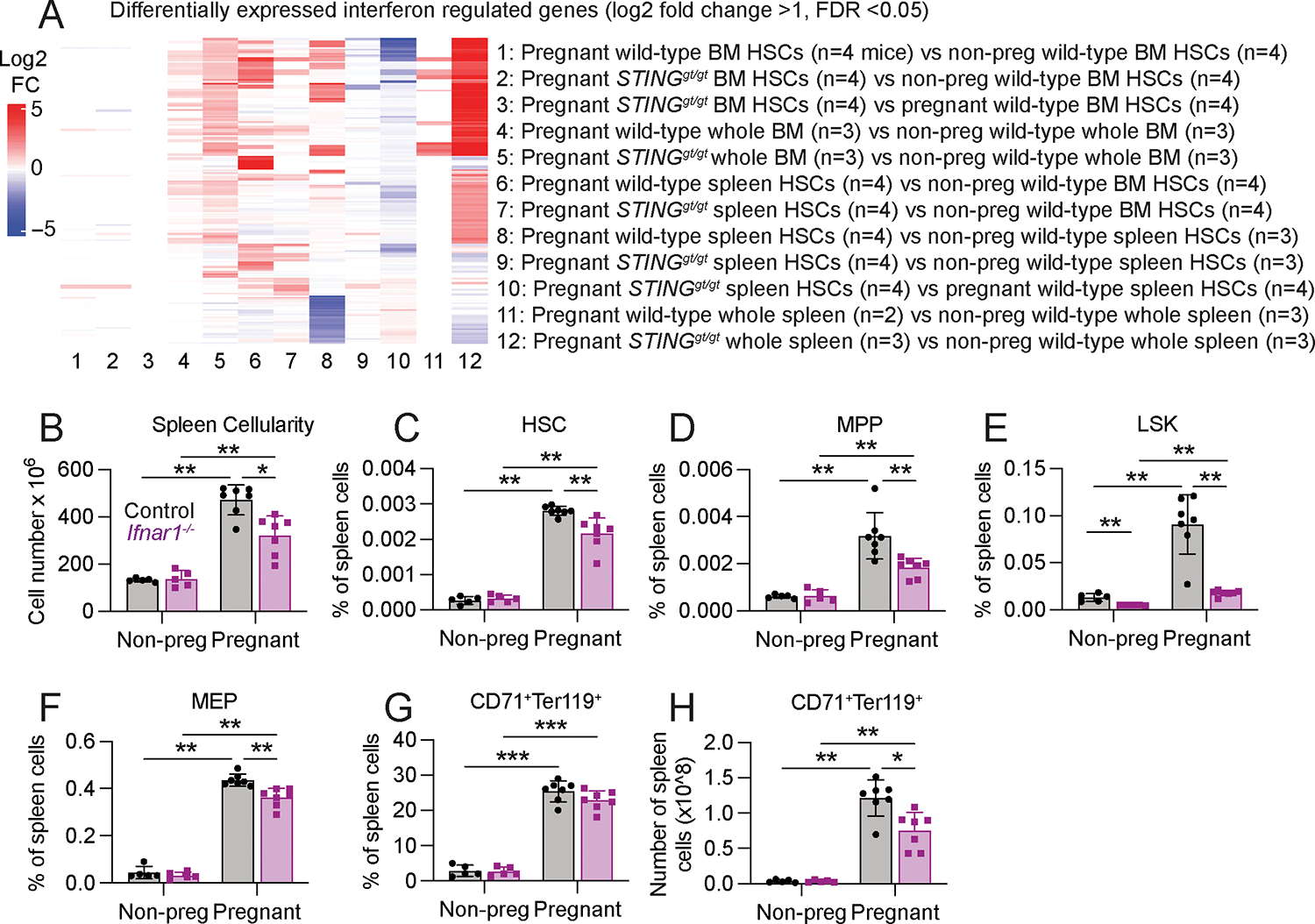
STING-dependent interferon expression increases during pregnancy and promotes splenic erythopoiesis. (**A**) Changes in interferon regulated gene expression among bone marrow and spleen HSCs as well as unfractionated bone marrow and spleen cells from pregnant and non-pregnant female *STING*^*gt/gt*^ and littermate wild-type mice. (**B-H**) Spleen cellularity (**B**) and the frequencies of HSCs (**C**), MPPs (**D**), LSK cells (**E**), MEPs (**F**), and CD71^+^Ter119^+^ erythroid progenitors (**G**) as well as the number of CD71^+^Ter119^+^ erythroid progenitors (**H**) in the spleen of pregnant or non-pregnant female *Ifnar1*^*−/−*^ or littermate control mice. Each dot represents a different mouse (5 to 7 mice per treatment in 4 independent experiments). The flow cytometry gates are shown in Fig. S2. All data represent mean ± standard deviation (*p < 0.05; **p < 0.01; ***p < 0.001). Statistical significance was assessed using Mann-Whitney tests followed by Holm-Sidak’s multiple comparisons adjustments **(B-F, H)**, or two-way ANOVAs followed by Sidak’s multiple comparisons adjustments (**G)**,

Given the increase in IFN levels during pregnancy, we assessed the expression of interferon regulated genes by RNA-sequencing. We observed changes in the expression of interferon regulated genes in splenic HSCs from pregnant mice as compared to splenic HSCs and bone marrow HSCs from non-pregnant mice (columns 6 and 8 in Fig. 5A). Some interferon regulated genes increased in expression (red in Fig. 5A) and some decreased in expression (blue in Fig. 5A), as expected for interferon regulated genes (44, 45). *STING* deficiency largely eliminated these changes in HSCs from pregnant mice (columns 7 and 9 in Fig. 5A). We observed little change in the expression of interferon regulated genes in bone marrow HSCs from pregnant as compared to non-pregnant mice (columns 1 and 2 in Figure 5A). We observed more limited changes in the expression of interferon regulated genes in whole bone marrow and whole spleen cells from pregnant as compared to non-pregnant mice (columns 4 and 11 of Figure 5A). We observed larger changes in the expression of interferon regulated genes in whole bone marrow and whole spleen cells from pregnant *STING* deficient mice as compared to non-pregnant mice (columns 5 and 12 in Fig. 5A). *STING* deficient mice have defects in the expression of anti-inflammatory factors that exacerbate inflammation in some circumstances (46). Overall, the data suggest that STING is necessary in HSCs for most of the changes in interferon regulated gene expression that are induced by pregnancy, but that STING negatively regulates interferon responses in some other cells in the bone marrow and spleen.

While we did not observe an increase in IFNγ levels in plasma, bone marrow or spleen lysate during pregnancy, multiple gene sets related to type II interferon signaling were enriched in spleen HSCs from pregnant mice as compared to spleen HSCs from non-pregnant mice (Fig. S10D). IFNγ signaling thus increased in HSCs, but not systemically, during pregnancy.

Spleen HSCs from estradiol-treated mice and serially-bled mice, also showed changes in the expression of interferon regulated genes as compared to bone marrow and spleen HSCs from untreated control mice (Fig. S9G and S9H).

By RNA-sequencing, we did not detect the expression of interferon transcripts in HSCs or unfractionated cells from the bone marrow or spleen of pregnant or non-pregnant mice. Given that interferons can be difficult to detect by RNA-sequencing, we also assessed this by quantitative RT-PCR. By quantitative RT-PCR, we found that *Ifna* (which encodes IFNα) was elevated in HSCs from the bone marrow and spleen of pregnant mice as compared to HSCs in the bone marrow and spleen of non-pregnant mice (Fig. S11A and S11B). However, we did not detect increased *Ifna* expression in unfractionated cells, LK myeloid progenitors, CD3^+^ T cells, B220^+^ B cells, or Mac-1^+^Gr-1^+^ myeloid cells in the bone marrow or spleen of pregnant mice (Fig. S11A and S11B). The increase in *Ifna* expression in spleen HSCs during pregnancy was not observed in *STING*^*gt/gt*^ mice (Fig. S11C and S11D). These data suggest that cGAS-STING signaling promoted *Ifna* expression in HSCs during pregnancy.

### Type I interferon contributed to the increase in splenic erythropoiesis

To test if the effects of cGAS-STING signaling on HSCs and erythropoiesis during pregnancy were partly mediated by type I interferon I (IFNα or IFNβ), we examined *Ifnar1*^*−/−*^ mice, which lack type-I IFN receptor function (47). In non-pregnant female mice, *Ifnar1* deficiency did not affect blood cell counts (Fig. S11E–G), bone marrow (Fig. S11H) or spleen (Fig. 5B) cellularity, or the frequencies of HSCs, MPPs, MEPs, or CD71^+^Ter119^+^ erythroid progenitors in the bone marrow (Fig. S11I–M) or spleen (Fig. 5C–H). In pregnant mice, *Ifnar1* deficiency reduced spleen cellularity (Fig. 5B) and the frequencies of HSCs, MPPs, LSK cells, and MEPs in the spleen (Fig. 5C–F). *Ifnar1* deficiency did not reduce the frequency of CD71^+^Ter119^+^ erythroid progenitors in the spleen (Fig. 5G) but it did reduce the absolute number of CD71^+^Ter119^+^ erythroid progenitors (Fig. 5H), consistent with the reduction in spleen cellularity (Fig. 5B). Type I interferon, thus, contributed to the increase in splenic erythropoiesis during pregnancy. We did not observe a decline in red blood cell counts in *Ifnar1*-deficient mice (Fig. S11F), indicating that other mechanisms also contributed to the effects of cGAS-STING signaling on erythropoiesis during pregnancy, potentially including type II interferon, which can be induced by type I interferon (48).

### Retrotransposons were derepressed in HSCs during pregnancy in humans

**Fig. 6:**
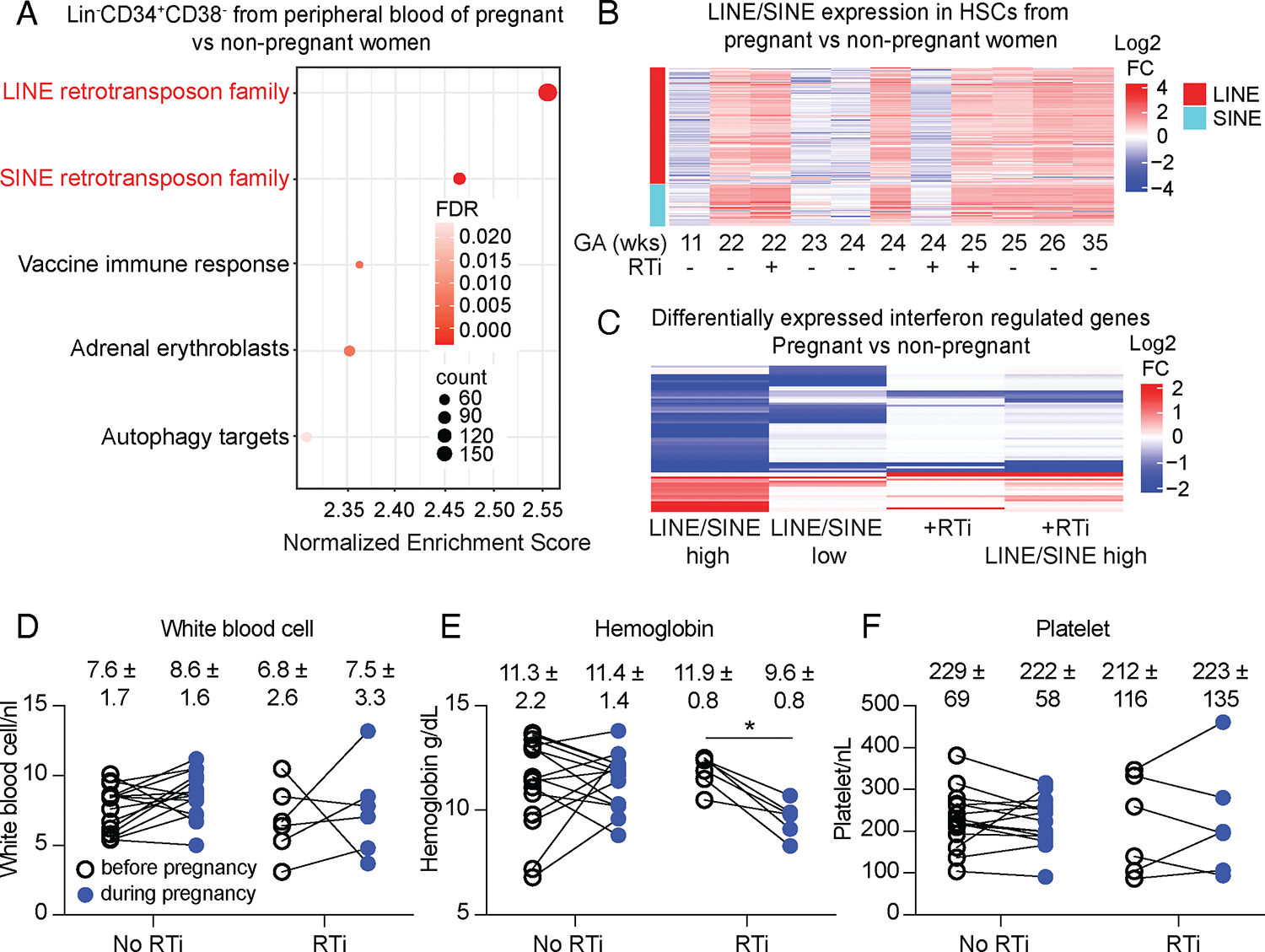
Retrotransposon expression is increased in HSCs during pregnancy in humans and was associated with the development of anemia. **(A**) Gene set enrichment analysis on RNA sequencing data from Lin^−^CD34^+^CD38^−^ cells isolated from the blood of pregnant and non-pregnant females. (**B**) Changes in retrotransposon expression in Lin^−^CD34^+^CD38^−^ cells from the blood of pregnant females (n=11; 3 of whom were taking reverse transcriptase inhibitors; GA means gestational age) as compared to average values from Lin^−^CD34^+^CD38^−^ cells obtained from non-pregnant females (n=3). (**C**) Differential expression of interferon regulated genes in Lin^−^CD34^+^CD38^−^ cells from pregnant as compared to non-pregnant females. HSCs were categorized based on high LINE/SINE expression and no reverse transcriptase inhibitor treatment (column 1), low LINE/SINE expression and no reverse transcriptase inhibitor treatment (column 2), all reverse transcriptase inhibitor treated without regard to LINE/SINE expression (column 3), and reverse transcriptase inhibitor treated and high LINE/SINE expression (column 4). **(D-F)** Blood cell counts from individuals with and without reverse transcriptase inhibitor treatment, before and during pregnancy, lines connect the same individual. The flow cytometry gates are shown in Fig. S12. All data represent mean ± standard deviation (*p < 0.05; **p < 0.01; ***p < 0.001). Statistical significance was assessed using paired *t*-tests followed by Holm Sidak’s multiple comparisons adjustments **(D - F)**. All statistical tests were two-sided. RTis: reverse transcriptase inhibitors.

To test if retrotransposons are derepressed in humans during pregnancy, we performed RNA sequencing on Lineage^−^CD34^+^CD38^−^ cells, which are highly enriched for HSCs (49), from the blood of pregnant and non-pregnant females. The two most highly enriched gene sets in HSCs from pregnant as compared to non-pregnant females were LINE and SINE retrotransposons (Fig. 6A) - the most active retrotransposon families in humans (50). The third and fourth most highly enriched gene sets related to a vaccine immune response, including several interferon regulated genes (51), and erythropoiesis (52) (Fig. 6A). During pregnancy, the increase in retrotransposon transcription in HSCs did not occur uniformly, as approximately one-third of HSC samples (each from a different person) did not exhibit clear increases in retrotransposon transcription (Fig. 6B). HSCs with higher LINE and SINE expression had more pronounced changes in the expression of interferon regulated genes than those with low LINE and SINE transcription (Fig. 6C). Human HSCs thus also exhibited increased retrotransposon transcription and changes in the expression of interferon regulated genes during pregnancy.

We also performed RNA sequencing on Lineage^−^CD34^+^CD38^−^ cells from the blood of three pregnant people with HIV who were undergoing treatment with reverse transcriptase inhibitors. HIV viral load was undetectable in these individuals, indicating it was well controlled (53). HSCs from two of the people exhibited increased transcription of retrotransposons relative to HSCs from non-pregnant females (Fig. 6B). However, the HSCs from pregnant females taking reverse transcriptase inhibitors exhibited many fewer changes in the expression of interferon regulated genes as compared to HSCs from females not taking reverse transcriptase inhibitors, even when only HSCs with strong LINE and SINE expression were considered (Fig. 6C).

We retrospectively assessed blood cell counts before and during pregnancy in 15 individuals who did not take reverse transcriptase inhibitors and 6 who did. Those who were not taking reverse transcriptase inhibitors did not exhibit statistically significant changes in white blood cell or platelet counts or hemoglobin levels during pregnancy as compared to before pregnancy (Fig. 6D–F). Individuals taking reverse transcriptase inhibitors did not exhibit changes in white blood cell or platelet counts during pregnancy (Figure 6D and 6F); however, they did exhibit declines in hemoglobin levels during pregnancy, such that they all became anemic (Figure 6E). These data suggested that humans also derepress retrotransposons during pregnancy to activate innate immune pathways in HSCs and to promote erythropoiesis.

## Discussion

Our findings show that retrotransposons are derepressed in HSCs during pregnancy, activating cGAS-STING signaling and triggering an interferon response that promotes HSC activation and increases erythropoiesis. Our data suggested that this is necessary to avoid anemia during pregnancy in mice and humans. The observation that retrotransposons were also derepressed in HSCs from serially-bled mice suggests that these mechanisms are broadly employed to increase HSC function and erythropoiesis in response to erythropoietic stresses. This is surprising given that retrotransposon expression declines in mature blood cells during pregnancy (54) and given the potential for retrotransposon-mediated mutagenesis (55); however, additional studies will be required to test if the derepression of retrotransposons in HSCs actually increases retrotransposon-mediated mutagenesis or if such mutations are propagated to differentiated hematopoietic cells.

Treatment with estradiol increased retrotransposon transcription and chromatin accessibility at loci that overlap with retrotransposons. However, many more retrotransposons increased in expression during pregnancy as compared to after estradiol treatment. Moreover, retrotransposons were also derepressed after serial bleeding, which would not be expected to be mediated by changes in estrogen. Therefore, there are likely to be additional, estrogen-independent, mechanisms that promote retrotransposon expression after erythropoietic stresses.

cGAS-STING-IFNα signaling does not appear to be entirely responsible for the effects of retrotransposons on erythropoiesis during pregnancy. cGAS-STING signaling may induce additional interferons beyond IFNα. cGAS-STING signaling may also have interferon-independent effects on HSCs and erythropoiesis, as cGAS-STING can also regulate cell death (56), proliferation (57), and autophagy (58) through interferon-independent mechanisms. It is possible that other innate immune pathways contribute to the effect of retrotransposons on HSCs and erythropoiesis.

In humans, females taking reverse transcriptase inhibitors exhibited lower hemoglobin levels during pregnancy, in contrast to those who did not take reverse transcriptase inhibitors. This is consistent with a report that people with HIV who take reverse transcriptase inhibitors are much more likely to become anemic during pregnancy than those who do not (59). HIV disease may contribute to anemia, but the individuals in our study were asymptomatic with well-controlled disease and undetectable viral loads. Non-pregnant, asymptomatic, HIV-infected females have a similar incidence of anemia as the general population (60). Our data raise the possibility that reverse transcriptase inhibitor use promotes the development of anemia in pregnant humans by inhibiting the activation of innate immune pathways by retrotransposons; however, additional studies will be required in larger numbers of patients from more institutions to evaluate this.

Commensal microbiota induce endogenous retroviruses in keratinocytes, activating cGAS/STING signaling, and recruiting T cells that promote immunity and wound repair (21). In light of this observation, our results raise the question of whether retrotransposons are commonly derepressed in stem cells after tissue injury and whether this contributes widely to tissue regeneration.

## Materials and Methods

### Reagents

The markers used for the identification of the cell populations examined in this study can be found in Table S2 and details related to antibodies and primer sequences can be found in Tables S3 and S4, respectively.

### Mice

All mouse experiments complied with all relevant ethical regulations and were performed according to protocols approved by the Institutional Animal Care and Use Committee at the University of Texas Southwestern Medical Center (protocol 2019–102632-G). *Vav1-iCre* (JAX stock #008610) (61) and *Ifnar1*^*−/−*^ (JAX stock #028288) (62) mice were obtained from Jackson Laboratory. *STING*^*gt/gt*^ mice (JAX stock #017537) *(40)* and *cGAS*^*fl/fl*^ mice were obtained from Zhijian Chen (UT Southwestern). All mice were analyzed between 7 and 16 weeks of age unless otherwise indicated. All mice were backcrossed at least four times onto a C57BL/Ka (CD45.2) background. All mice were analyzed between 7 and 16 weeks of age unless otherwise indicated. Most experiments involved only female mice, such as experiments involving pregnancy and bleeding. Experiments with reverse transcriptase inhibitors and *STING* and *cGAS* deficiency in non-pregnant conditions involved male and female mice. For transplantation assays, C57BL/Ka-Thy-1.1/C57BL/Ka-Thy-1.2 (CD45.2/CD45.2) mice were used as recipients and C57BL/Ka-Thy-1.2 (CD45.1) mice were used as a source of competitor bone marrow cells. Mice were housed at the Animal Resource Center at the University of Texas Southwestern Medical Center in AAALAC-accredited, specific-pathogen-free animal care facilities under a 12 h:12 h light:dark cycle with a temperature of 18–24 °C and humidity of 35–60%. Mice were housed with a maximum of 5 same-sex mice per cage. Breeding cages had one male and two females. Igloos and bedding were placed in each cage for enrichment. Mice were fed normal chow (Teklad Global 16% Protein Rodent Diet; Envigo #2016). Mice were euthanized using isoflurane, and cervical dislocation was used as a secondary method of euthanasia.

### RNA sequencing in mouse hematopoietic cells

Cells were sorted into RLT buffer plus (Qiagen RNeasy Plus Micro kit) and RNA was purified according to the manufacturer’s instructions. RNA quality was validated using a Pico Bioanalyzer. Libraries were generated using SMARTer Stranded Total RNA-Seq kit – v2 Pico Input Mammalian (Takara). Library fragment size was measured using D1000 Screen Tape (Agilent) and libraries were quantified using the Qubit dsDNA high-sensitivity assay kit (Life Technologies). Libraries were sequenced using an Illumina NextSeq 500 with 200 base pair paired-end sequencing. The quality of RNA-seq raw reads was checked using FastQC 0.11.8. Raw reads were trimmed using TrimGalore 0.6.4 and mapped to the Ensembl GRCm38 mouse reference genome using STAR2.7.9a. Mapped reads were quantified using TETranscripts 2.2.1. Quantified mapped reads were normalized using DESeq2 1.30.0 with R 4.0.2. Differential expression was assessed using DESeq 2 1.30.0. Gene set enrichment analyses were performed using GSEA 4.1.0. Dot plots showing gene set enrichment analyses were made in R using the ggplot2 package. Venn diagrams showing the overlap in retrotransposons that were upregulated under various conditions were made using DeepVenn (63). Heatmaps showing the fold-change of retrotransposons or interferon regulated genes that were differentially expressed among conditions or cells were made in R using the ggplots2 and pheatmap packages. The heatmaps in Fig. 5A, S8G, and S8H show all mouse interferon regulated genes (based on interferome.com (64)) that were differentially expressed (log2 fold change >1, FDR <0.05) among cells. The heatmap in Fig. 5K shows all human interferon response genes (64) that were differentially expressed among samples.

Retrotransposon gene sets were from TETranscripts 2.2.1 while all other gene sets were from the Molecular Signature Database (MSigDB). In Figure 1A, the ECM organization gene set was annotated as “Reactome extracellular matrix organization” in MSigDB, the ECM structure gene set was annotated as “GOMF extracellular matrix structural constituent” in MSigDB, the EMT transition gene set was annotated as “Hallmark epithelial mesenchymal transition” in MSigDB, the ECM proteoglycan gene set was annotated as “Reactome ecm proteoglycans” in MSigDB, and the ECM degradation gene set was annotated as “Reactome degradation of the extracellular matrix” in MSigDB.

### ATAC sequencing

Three replicates of 1000 CD150^+^CD48^−^Lineage^−^Sca-1^+^c-kit^+^ cells per replicate were isolated by flow cytometry from different mice and sorted into MACS freezing solution (Milentenyi Biotec). Libraries for ATAC sequencing were prepared as described (65). The libraries were sequenced using 200 base pair (bp) paired-end reads with an Illumina NextSeq 500. Adapter sequences were trimmed using Cutadapt 2.5. Bowtie2 2.1.0 was used to align reads to the Ensembl GRCm38 mouse reference genome. Samtools 1.18 was used for data filtering and file format conversion. Blacklist regions and mitochondrial DNA were removed before peak calling. The genomic location of peaks was determined using Genrich 0.6.1. Differentially accessible peaks were identified using DiffBind 3.2.5 with default parameters. Peaks that were enriched (fold change > 2, FDR <0.05) were considered to gain accessibility. Peaks were considered to overlap with genes or retrotransposons if at least part of the peak fell between 1000 bp upstream of the transcription start site and the end of the gene or retrotransposon.

### Serial bleeding

Two to three month old female mice were bled via the tail vein five times over a 12 day period, removing approximately 200 ul of blood each time. Mice were analyzed 2 days after the last bleed.

### Drug treatments

Mice were treated with estradiol by injecting subcutaneously with 2 μg/day E2 (Sigma-Aldrich) in corn oil (Sigma-Aldrich). Mice were analyzed 1 day after the last injection. In Figure 1D–F, male mice were injected with E2 for 6 days and male controls were injected with corn oil. In Figure 1G–H, female mice were injected for 13 days. To treat with reverse transcriptase inhibitors, we made a stock solution in PBS that contained 12.5 mg/ml of tenofovir disproxil fumarate and 7.5 mg/ml of emtricitabine (both from ACROS organics). We then administered to mice by gavaging with this solution daily for 5 days such that the mice received 100 mg/kg body mass/day of tenofovir and 60 mg/kg/day of emtricitabine (21).

### Flow cytometric analysis and sorting of mouse hematopoietic cells

Bone marrow was flushed from one tibia and one femur using staining medium (Ca^2+^- and Mg^2+^-free Hank’s balanced salt solution (HBSS) supplemented with 2% heat-inactivated bovine serum). Spleens were mechanically dissociated by crushing them between two glass slides. The cells were dissociated into single cell suspensions by gently passing through a 25-gauge needle and then filtering through 70-μm nylon mesh. Cells were counted, and stained with antibodies by incubating cell suspensions on ice for 30 min. For analysis of HSCs, cells were stained with fluorophore-conjugated antibodies against lineage markers (CD2, CD3, CD5, CD8a, Gr1, Ter119 and B220), as well as c-kit, Sca-1, CD150 and CD48 (see Table S3 for details related to antibodies). For analysis of restricted progenitors, the cells were stained with fluorophore-conjugated antibodies against lineage markers, c-kit, Sca-1, CD16/32, CD34, CD105, and CD150. For analysis of differentiated cells, cells were stained with fluorophore-conjugated antibodies against Mac-1, Gr-1, B220, CD3, Ter119 and CD71. Cells were analyzed using a FACS Canto RUO (BD Biosciences) or a FACS Lyric (BD Biosciences) cytometer. Dead cells were identified and gated out of all analyses by including 1 μg/ml 4′,6-diamidino-2-phenylindole (DAPI) or propidium iodide in the staining medium used to resuspend cells for flow cytometry. Flow cytometry data were analyzed using Flowjo (BD Biosciences). The markers used to identify cell populations are summarized in Table S2 and the flow cytometry gates are shown in Figs. S1 and S2.

### Competitive reconstitution assays

Recipient mice (CD45.1/CD45.2) were irradiated using an XRAD 320 X-ray irradiator (Precision X-Ray) with two doses of 540 rad delivered at least 3 hours apart. For bone marrow transplantation, 5 × 10^5^ bone marrow cells from donor (CD45.2) mice and 5 × 10^5^ bone marrow cells from competitor mice (CD45.1) were mixed and injected intravenously through the retro-orbital venous sinus into recipient mice (CD45.2/CD45.1). For spleen transplantation, 1.5 × 10^6^ spleen cells from donor (CD45.2) and 3 × 10^5^ bone marrow cells from competitor mice (CD45.1) were mixed and injected intravenously through the retro-orbital venous sinus into recipient mice (CD45.2/CD45.1). For spleen transplantation from pregnant donors, 1.5 × 10^6^ spleen cells from the donor (CD45.2) and 5 × 10^5^ bone marrow cells from competitor mice (CD45.1) were mixed and injected intravenously into recipient mice (CD45.2/CD45.1).

Every 4 weeks until 16 weeks after transplantation, 50 to 100 μl of blood was collected from the tail vein and mixed with 200 μl of 10 mM EDTA in PBS to prevent clotting. Cells were subjected to ammonium-chloride potassium chloride red cell lysis. Cells were stained with antibodies against CD45.1 (A20, Biolegend #110706), CD45.2 (104, Tonbo #75–0454), Mac-1 (M1/70, Thermo #47–0112-82), Gr-1 (RB6–8C5, Tonbo #60–5931), B220 (RA3–6B2, Tonbo #20–0452) and CD3 (17A2, Biolegend #100206) to evaluate the frequencies of donor Mac-1^+^Gr-1^+^ myeloid cells, B220^+^ B cells, and CD3^+^ T cells. All antibodies were used at 1:200 dilution and cells were stained for 30 min on ice.

### BrdU incorporation

Mice were intraperitonially injected with 0.1 mg/g body mass of BrdU dissolved in PBS and maintained on 1 mg/ml BrdU in the drinking water for 72 hours for HSC analysis in non-pregnant mice and 24 hours for HSCs in pregnant mice. Bone marrow cells were obtained from the spine and legs and c-kit^+^ cells were enriched as described above. HSCs were double sorted into a 1.5 ml tube containing 2 × 10^6^ carrier bone marrow cells from a mouse not treated with BrdU. The cells were then stained for BrdU using the BD APC BrdU Flow Kit following the manufacturer’s instructions. HSCs were distinguished from unstained carrier cells based on c-kit staining. BrdU levels in HSCs were analyzed using a FACSAria II cytometer. The markers used to identify HSCs are summarized in Table S2 and the flow cytometry gates are shown in Figs. S1 and S2.

### Immunofluorescence and confocal imaging

HSCs were double sorted into PBS and allowed to adhere to poly-L-lysine coated cover slips (Corning) for 30 minutes at 4°C. Cells were then fixed with 4% paraformaldehyde for 15 minutes and then permeabilized in PBS-0.5% Triton X-100 for 5 minutes, washed 3 times with PBS, then blocked for 45 minutes in PBS containing 3% BSA (PBS-BSA) and incubated with primary antibody diluted in PBS-BSA for 60 minutes. Cells were then washed 3 times with PBS-0.05% Triton X-100 and incubated with secondary antibody in PBS-BSA for 60 minutes, then washed 3 times with PBS-0.05% Triton X-100. Following primary and secondary antibody incubation, cells were incubated with Dylight phalloidin (to stain F-actin; 13054S, Cell Signaling Technology, 1:200) in PBS for 15 minutes, then rinsed 3 times with PBS. Cells were incubated in DAPI (4ug/ml) to visualize nuclei. ProLong Gold Antifade (Fisher Scientific) was used for mounting. Images were acquired using a Nikon W1 spinning disk confocal microscope at 100x. The primary antibody was anti-phosphoSTING. Secondary antibody was goat anti-rabbit Alexa Fluor 647. For quantification of confocal immunofluorescence images, an automated macro was developed and implemented using FIJI to facilitate the analysis of the image data. A maximum intensity (MAXI) projection was generated of the Z-stack images which were obtained to cover the entire volume of the cells. Phalloidin staining was used to determine cell size/shape and to define the region of interest (ROI) for each cell. Within these ROIs, the macro calculated the total integrated density of the fluorescence signal from the protein of interest (phosphoSTING). Background fluorescence, measured in an area with no cells, was subtracted to correct for non-specific signals. The total integrated density was normalized against cell size.

### Measurement of cytokines

Blood plasma was obtained after centrifugation at 900×g for 10 minutes. Bone marrow plugs and spleens were lysed in protein lysis buffer (100 mM Tris, 150 mM NaCl, 1 mM EGTA, 1 mM EDTA, 1% Triton and 0.5% sodium deoxycholate). Cytokines were measured using PBL high-sensitivity IFNα mouse ELISA kit and Biolegend LEGENDplex (Biolegend Mouse Inflammation Panel #740446) according to the manufacturer’s instructions. Data were analyzed using the LEGENDplex Data Analysis Software. All samples were analyzed in duplicate.

### RNA extraction and real-time qPCR from mouse hematopoietic cells

For RNA extraction from sorted hematopoietic cells, 500 to 1,000 splenic HSCs or 2,000 to 10,000 cells of all other cell types were double sorted into 300 μl of buffer RLT plus (Qiagen RNeasy Plus Micro kit) and purified according to the manufacturer’s instructions. RNA was reverse transcribed using iScript Reverse Transcription Supermix (Bio-Rad). Transcript levels were normalized to *Actb* and fold change was calculated using the ΔCt method.

### Sorting of human hematopoietic cells

Blood was collected into S-Monovette EDTA tubes from healthy pregnant females between 11 and 35 weeks of gestation with no evidence of hematopoietic malignancies. Peripheral blood mononuclear cells (PBMCs) were isolated from the samples via density gradient centrifugation (Biocoll, Bio X Cell, #BS.L6115) for 30 minutes at 1400 rpm at room temperature. The PBMC fraction was washed with PBS and the cells were resuspended in PBS supplemented with 10% FBS. Total PBMC number and PBMC viability was determined for each sample. PBMCs were stained with directly conjugated antibodies against human CD3, CD4, CD8, CD14, CD235, CD56, CD34, CD38 and the viability dye SYTOX Blue (Invitrogen, S11348) for 30 minutes on ice. Cells were then washed and resuspended in staining medium (PBS supplemented with 5% FBS). Lineage^−^CD34^+^CD38^−^ cells, which are highly enriched for HSCs, were isolated using a FACSAria III flow cytometer. The markers used to identify human Lineage^−^CD34^+^CD38^−^ cells are summarized in Table S2 and the flow cytometry gates are shown in Fig. S12. Analysis of human samples was conducted in accordance with the local ethics committee at the West German Biobank Essen (No.: 12–5212-BO). Informed consent was obtained from study participants. Samples were collected from females whose age ranged from 27 to 40 years.

### RNA sequencing using human hematopoietic cells

Cells were sorted into RLT buffer (Qiagen RNeasy Mini kit) and RNA was purified according to the manufacturer’s instructions. RNA quality was validated using a Pico Bioanalyzer. Libraries were generated with Watchmaker RNA Library Prep Kit (Watchmaker Genomics Limited, London, UK) using xGen Stubby Adapter-UDI Primers (IDT, Coralville, IA, USA). Libraries were quantified using the Qubit dsDNA high sensitivity-assay kit (Invitrogen, Waltham, MA, USA) and Agilent Bioanalyzer DNA chips. Libraries were sequenced using an Illumina NextSeq 2000 with 300 base pair paired-end sequencing. The quality of RNA-seq raw reads was checked using FastQC 0.11.8. Raw reads were trimmed using TrimGalore 0.6.4 and mapped to the Ensembl GRCh38 human reference genome using STAR2.7.9a. Mapped reads were quantified using TETranscripts 2.2.1. Quantified mapped reads were normalized using DESeq2 1.30.0 with R 4.0.2. Differential expression was assessed using DESeq2 1.30.0. Gene set enrichment analyses were performed using GSEA 4.1.0. Dot plots showing gene set enrichment analyses were made in R using the ggplot2 package. Heat maps showing the fold-change of deregulated genes were made in R using the ggplots2 and pheatmap packages. Retrotransposon gene sets were from TETranscripts 2.2.1 while all other gene sets were from the Molecular Signature Database (MSigDB). In Figure 5I, the Vaccine immune response gene set was annotated as “Hoek B cell 2011 2012 tiv adult 7dy up” in MSigDB, the Adrenal erythroblast gene set was annotated as “DESCARTES adrenal erythroblasts” in MSigDB, and the Autophagy targets gene set was annotated as “Cadwell Atg16l1 targets up” in MSigDB.

### Peripheral blood cell counts in humans

Blood cell counts were acquired at the University Hospital Essen by retrospectively reviewing the electronic medical records of eligible participants. We assembled a list of females ranging in age from 25 to 36 who had routine blood draws during pregnancy over the last five years at the University Hospital Essen and then reviewed their medical histories to exclude those with a history of hematological malignancies. We identified the subset of these individuals who had routine blood draws at least one month before and two months after gestational day 1. To identify a cohort of people that were HIV positive, pregnant and taking reverse transcriptase inhibitors, we applied the same set of criteria except that patients were additionally screened for an undetectable HIV viral load, as well as reverse transcriptase inhibitor use beginning at least one year prior to becoming pregnant. Patient records were reviewed to assess white blood cell counts, hemoglobin, platelet counts, and HIV viral load (when applicable) before and during pregnancy. A retrospective review of patient records was conducted in accordance with local ethics committee approval at the University Hospital Essen (No.: 24–11998-BO). As the retrospective analyses of medical records did not require interactions or interventions with patients, and the data were shared in an anonymized manner, the Institutional Review Board waived the need to obtain signed informed consent from patients at the time of this data collection.

### Statistical methods

Mice were allocated to experiments randomly and samples processed in an arbitrary order, but formal randomization techniques were not used. No formal blinding was applied when performing the experiments or analyzing the data. Sample sizes were not pre-determined on the basis of statistical power calculations but were based on our experience with these assays. No data were excluded. Different replicates typically reflected samples obtained from different mice, although in competitive transplant assays the same mice were repeatedly bled at different timepoints from 4 to 16 weeks after transplantation.

Before analyzing the statistical significance of differences among groups, we tested whether data were normally distributed and whether variance was similar among groups. To test for normality, we performed Shapiro–Wilk tests when 3 ≤ *n* < 20 or D’Agostino Omnibus tests when *n* ≥ 20. To test whether variability significantly differed among groups, we performed *F*-tests (for experiments with two groups) or Levene’s median tests (for experiments with more than two groups). When the data significantly deviated from normality or variability significantly differed among groups, we log_2_ transformed the data and tested again for normality and variability. If the transformed data no longer significantly deviated from normality and equal variability, we performed parametric tests on the transformed data. If log_2_ transformation was not possible or the transformed data still significantly deviated from normality or equal variability, we performed non-parametric tests on the non-transformed data. The dagoTest and shapiroTest functions of the fBasics package in R were used to perform the normality tests, and the leveneTest function of the car package in R was used to perform the Levene’s median test for variances.

When data or log_2_-transformed data were normally distributed and equally variable, statistical analyses were performed using Student’s *t*-tests or paired *t*-tests (two groups), one-sample *t*-tests (when there was only one group), one-way analyses of variance (ANOVAs) (when there were more than two groups), two-way ANOVAs or matched samples two-way ANOVAs (when there were two or more groups with multiple tissues). When the data or log_2_-transformed data were normally distributed but unequally variable, statistical analyses were performed using Welch’s *t*-tests (when there were two groups). When the data and log_2_-transformed data were abnormally distributed or unequally variable, statistical analysis was performed using Mann–Whitney tests (when there were two groups), Kruskal–Wallis tests (when there were more than two groups), Friedman tests (when there were more than two paired groups), or nparLD tests for the overall differences between transplants and Mann-Whitney tests for the differences within time points (when there were two groups measured at multiple timepoints). *P* values from multiple comparisons were adjusted using Sidak’s method after ANOVAs or Dunn’s method after Kruskal–Wallis tests or Friedman tests. Holm–Sidak’s method was used to adjust comparisons involving multiple Student’s *t*-tests, Welch’s *t*-tests, paired *t*-tests, Mann–Whitney tests, or nparLD tests. All statistical tests were two-sided. All data represent mean ± standard deviation. Statistical tests were performed using GraphPad Prism V10.1.2 or R 4.0.2.

## Supplementary Material

1

## Data Availability

All data needed to evaluate the conclusions in the paper are available in the manuscript or in supplementary materials. All RNA and ATAC sequencing data have been deposited in the NCBI Sequence Read Archive (Bioproject ID PRJNA1142167).
