# Supplementary material for: Retrotransposons are co-opted to activate hematopoietic stem cells and erythropoiesis
Source: Science. Author manuscript; Available in PMC 2025 Jan 8. (PMC11709122; doi:10.1126/science.ado6836)
Supplement: 1 [file NIHMS2042983-supplement-1.pdf]

Science
AAAS

Supplementary Materials for

Retrotransposons are co-opted to activate hematopoietic stem cells and erythropoiesis

Julia Phan[1], Brandon Chen[1], Zhiyu Zhao[1], Gabriele Allies[2], Antonella Iannaccone[3], Animesh Paul[1], Feyza Cansiz[2], Alberto Spina[3], Anna-Sophia Leven[2], Alexandra Gellhaus[3], Dirk Schadendorf[2], Rainer Kimmig[3], Marcel Mettlen[4], Alpaslan Tasdogan[2*], Sean J. Morrison[1,5 *]

*Co-corresponding authors: Sean.Morrison@UTSouthwestern.edu and Alpaslan.Tasdogan@uk-essen.de

The PDF file includes:

Figs. S1 to S12

Tables S1 to S4

References *(66-69)*

Supplementary Figures

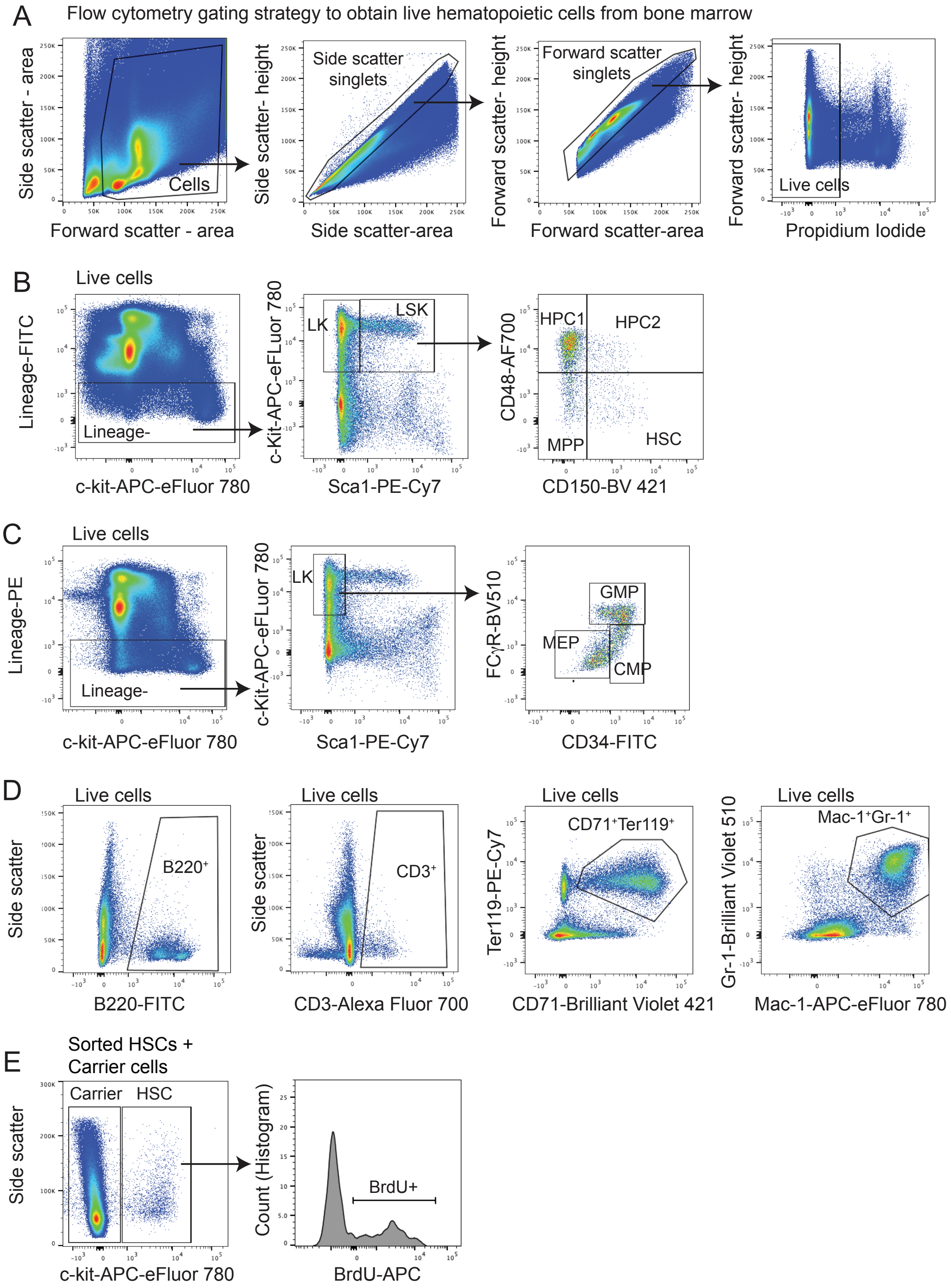

Fig. S1: Flow cytometry gating strategy for the isolation of hematopoietic stem and progenitor cells from mouse bone marrow. Representative flow cytometry gates used to identify hematopoietic stem and progenitor cell populations (**A-C**), as well as $B220^{+}$ B cells, $CD3^{+}$ T cells, $CD71^{+}Ter119^{+}$ erythroid cells, and $Mac\text{-}1^{+}Gr\text{-}1^{+}$ myeloid cells (**D**) from the bone marrow. **(E)** Representative flow cytometry gates used to identify BrdU+ HSCs from the bone marrow. The markers used to identify each of the cell populations characterized in this study are listed in Supplementary Table 1.

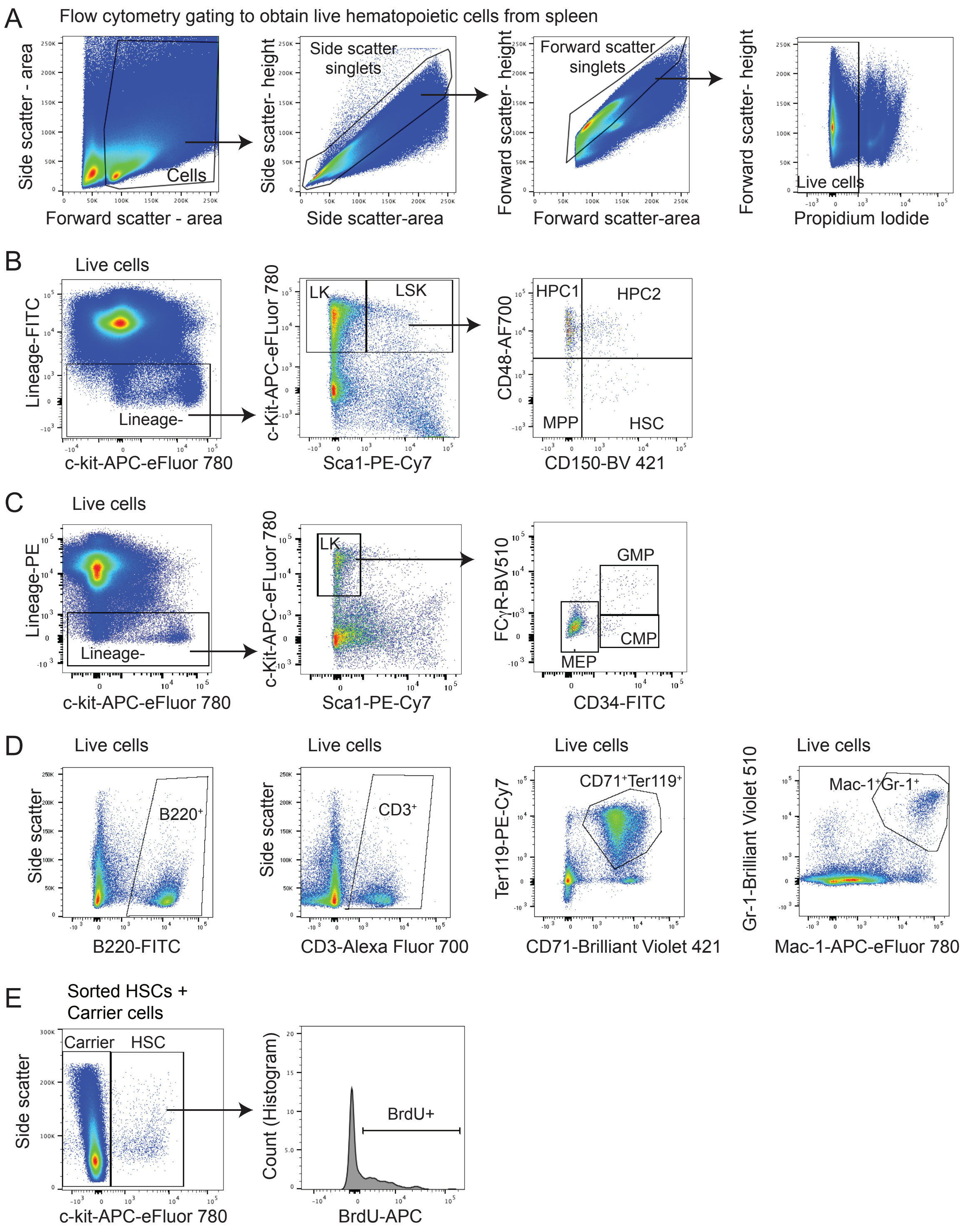

Fig. S2: Flow cytometry gating strategy for the isolation of hematopoietic stem and progenitor cells from mouse spleen. Representative flow cytometry gates used to identify hematopoietic stem and

progenitor cell populations (**A-C**), as well as B220^{+} B cells, CD3^{+} T cells, CD71^{+}Ter119^{+} erythroid cells, and Mac-1^{+}Gr-1^{+} myeloid cells D) from the spleen. **(E)** Representative flow cytometry gates used to identify BrdU+ HSCs from the spleen. These samples were from the spleens of pregnant mice. The markers used to identify each of the cell populations characterized in this study are listed in Supplementary Table 1.

A

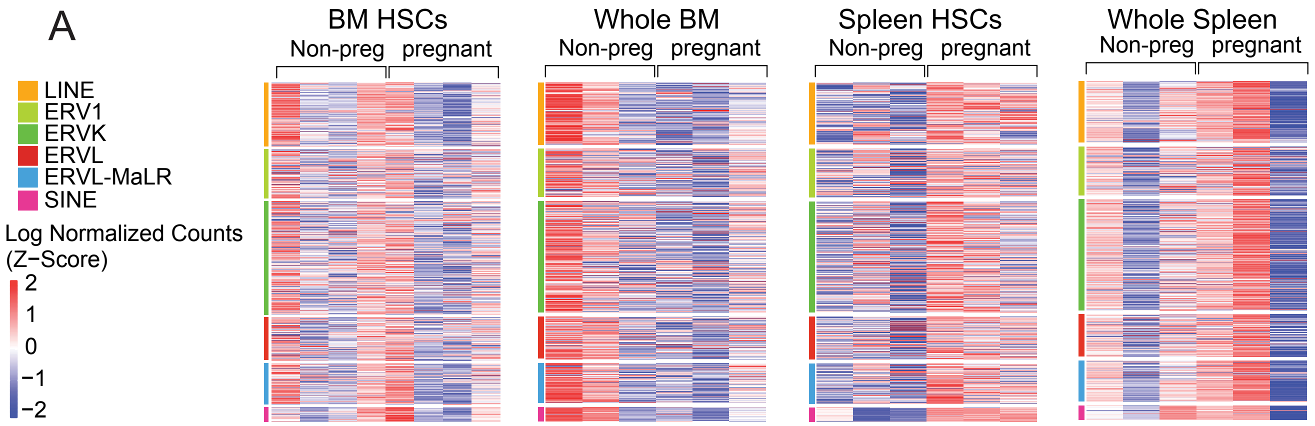

B

Retrotransposons families enriched in spleen HSCs from pregnant mice vs spleen HSCs from non-pregnant mice (NES>1.5, FDR<0.01)

	NES	FDR	Number of Retrotransposons Upregulated in Gene Set	Number of Retrotransposons in Gene Set
SINE retrotransposon family	2.21	0.000	32	36
LINE retrotransposon family	2.13	0.000	75	153
ERVK retrovirus family	1.90	0.001	110	275
ERVL-MaLR retrovirus family	1.85	0.003	52	99

C

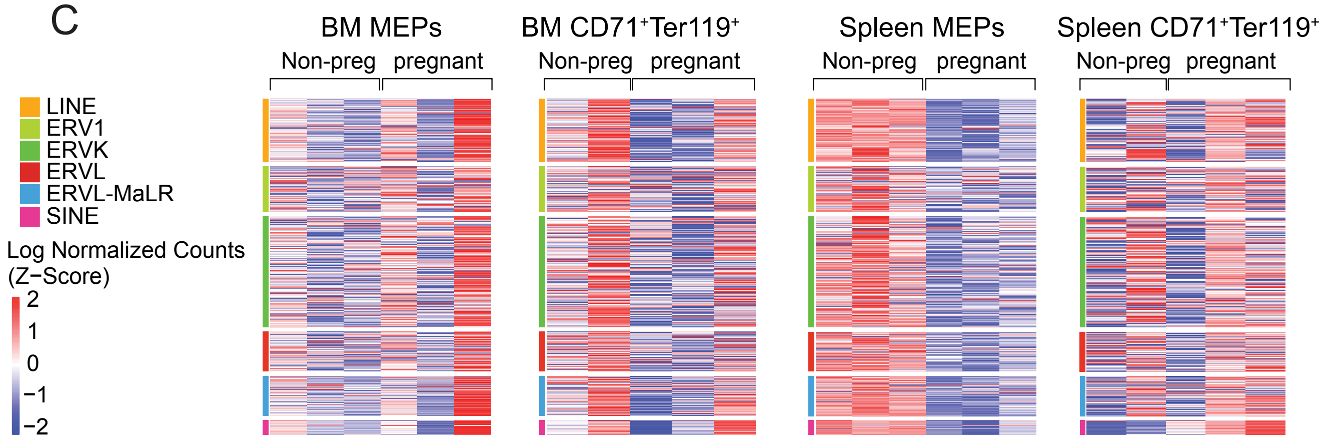

D

Retrotransposons families enriched in bone marrow MEPs from pregnant mice vs bone marrow MEPs from non-pregnant mice (NES>1.5, FDR<0.01)

	NES	FDR	Number of Retrotransposons Upregulated in Gene Set	Number of Retrotransposons in Gene Set
LINE retrotransposon family	1.99	0.000	86	153

Fig. S3: Retrotransposons are preferentially expressed in spleen HSCs during pregnancy. (A-D) We performed RNA sequencing on HSCs, MEPs, and $CD71^{+}Ter119^{+}$ cells and unfractionated cells from the bone marrow and spleen of pregnant and non-pregnant mice. **(A)** Z-score of log transformed normalized counts for retrotransposons in HSCs and unfractionated cells from the bone marrow and spleen of non-

pregnant and pregnant mice. Red means increased counts as compared to the average of all samples and blue means decreased counts. Each column represents a different mouse, with the exception of non-pregnant spleen HSCs, in which each column represents pooled samples from 7-12 mice. **(B)** Significantly enriched retrotransposon families in spleen HSCs from pregnant mice as compared to spleen HSCs from non-pregnant mice (NES>1.5, FDR<0.01). There were no significantly enriched retrotransposon families in bone marrow HSCs or unfractionated bone marrow or spleen cells from pregnant as compared to non-pregnant mice. (**C)** Z-score of log transformed normalized counts for retrotransposons in MEPs and $CD71^{+}Ter119^{+}$ cells from the bone marrow and spleen of non-pregnant and pregnant mice. Each column represents a different mouse. **(D)** Significantly enriched retrotransposon families in bone marrow MEPs from pregnant mice as compared to bone marrow MEPs from non-pregnant mice (NES>1.5, FDR<0.01). There were no significantly enriched retrotransposon families in MEPs from the spleen or $CD71^{+}Ter119^{+}$ cells from the bone marrow or spleen of pregnant mice as compared to non-pregnant mice.

A Top 10 upregulated gene sets in spleen HSCs from serially-bled mice as compared to spleen HSCs from normal mice

MSigDB Gene Set Annotation	NES	FDR	Number of Genes Upregulated in Gene Set	Number of Genes in Gene Set
Hallmark heme metabolism	1.81	0.000	105	179
Reactome RUNX1 regulates genes involved in megakaryocyte differentiation and platelet function	1.80	0.000	48	82
Chyla CBFA2T3 targets up	1.77	0.000	83	220
GOCC nucleosome	1.77	0.000	45	97
Reactome condensation of prophase chromosomes	1.77	0.000	43	60
Reactome HATs acetylate histones	1.76	0.000	36	72
Reactome PRC2 methylates histone and DNA	1.76	0.000	47	76
GOCC DNA packaging complex	1.75	0.000	55	126
Ivanova hematopoiesis mature cell	1.75	0.000	133	283
DESCARTES organogenesis primitive erythroid lineage	1.75	0.000	250	481

B Number of Differentially Expressed Retrotransposons (FC > 2, FDR < 0.05)

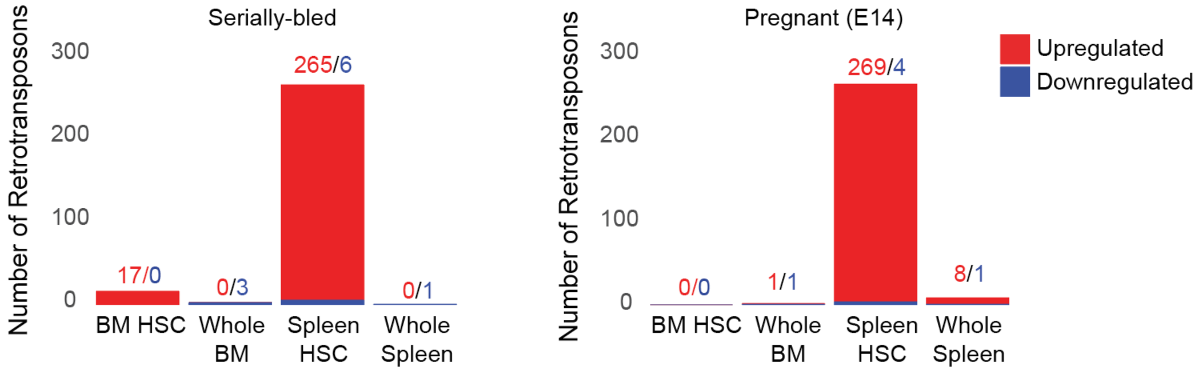

C Serially-bled

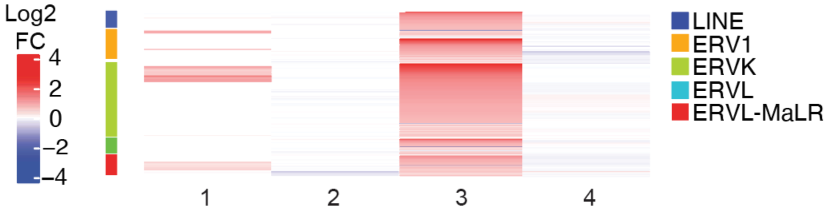

1: Serially-bled BM HSCs (n=3 mice) vs normal BM HSCs (n=3)
2: Serially-bled whole BM (n=2) vs normal whole BM (n=2)
3: Serially-bled spleen HSCs (n=2) vs normal BM HSCs (n=3)
4: Serially-bled whole spleen (n=2) vs normal whole spleen (n=3)

D Gene sets of retrotransposon families upregulated in splenic HSCs compared to bone marrow HSCs

Serially-bled

	NES	FDR
ERVK retrovirus family	2.15	0.000
ERV1 retrovirus family	1.94	0.000
LINE retroransposon family	1.85	0.000
ERVL-MaLR retrovirus family	1.81	0.000
ERVL retrovirus family	1.67	0.022

Pregnant (E14)

	NES	FDR
ERVK retrovirus family	2.44	0.000
ERV1 retrovirus family	2.12	0.000
LINE retroransposon family	2.07	0.001
ERVL-MaLR retrovirus family	2.07	0.003
ERVL retrovirus family	2.01	0.022

E Number of Upregulated Retrotransposons (FC>2, FDR<0.05)
Spleen HSCs vs Bone Marrow HSCs

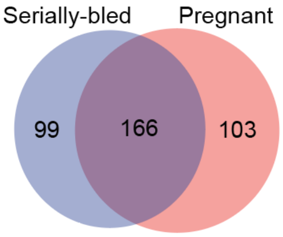

Fig. S4: Serial bleeding increases the transcription of retrotransposons in HSCs. We performed RNA sequencing on HSCs and unfractionated cells from the bone marrow and spleen of serially-bled and normal control mice. (**A**) Top 10 upregulated gene sets (NES>2, FDR<0.01) in splenic HSCs from serially-bled mice (n=2) as compared to splenic HSCs from normal mice (n=3, each replicate pooled from 7-12 mice). (**B**) Number of retrotransposons that significantly changed in expression (fold change > 2, FDR < 0.05) after serial bleeding or pregnancy in bone marrow HSCs (n=3 bleeding, n=4 pregnancy) or spleen HSCs (n=2 bleeding, n=3 pregnancy) as compared to control bone marrow HSCs (n=3 bleeding control, n=4 pregnancy control), or unfractionated bone marrow (n=2 bleeding vs 2 bleeding control, n=3 pregnancy vs 3 pregnancy control) or spleen cells (n=2 bleeding vs 3 bleeding control, n=2 pregnancy vs 2 pregnancy control) as compared to the same cell populations. **(C)** After serial bleeding, retrotransposons that significantly changed in expression (fold change > 2, FDR < 0.05) in bone marrow or spleen HSCs (as compared to control bone marrow HSCs) or unfractionated bone marrow or spleen cells. **(D)** Gene set enrichment analysis showed that ERV and LINE retrotransposon families were significantly enriched in splenic HSCs from serially-bled and pregnant mice as compared to bone marrow HSCs from control mice. (**E**) Overlap of retrotransposons that were upregulated (fold change >2, FDR <0.05) after serial bleeding and pregnancy in spleen HSCs as compared to bone marrow HSCs from control mice.

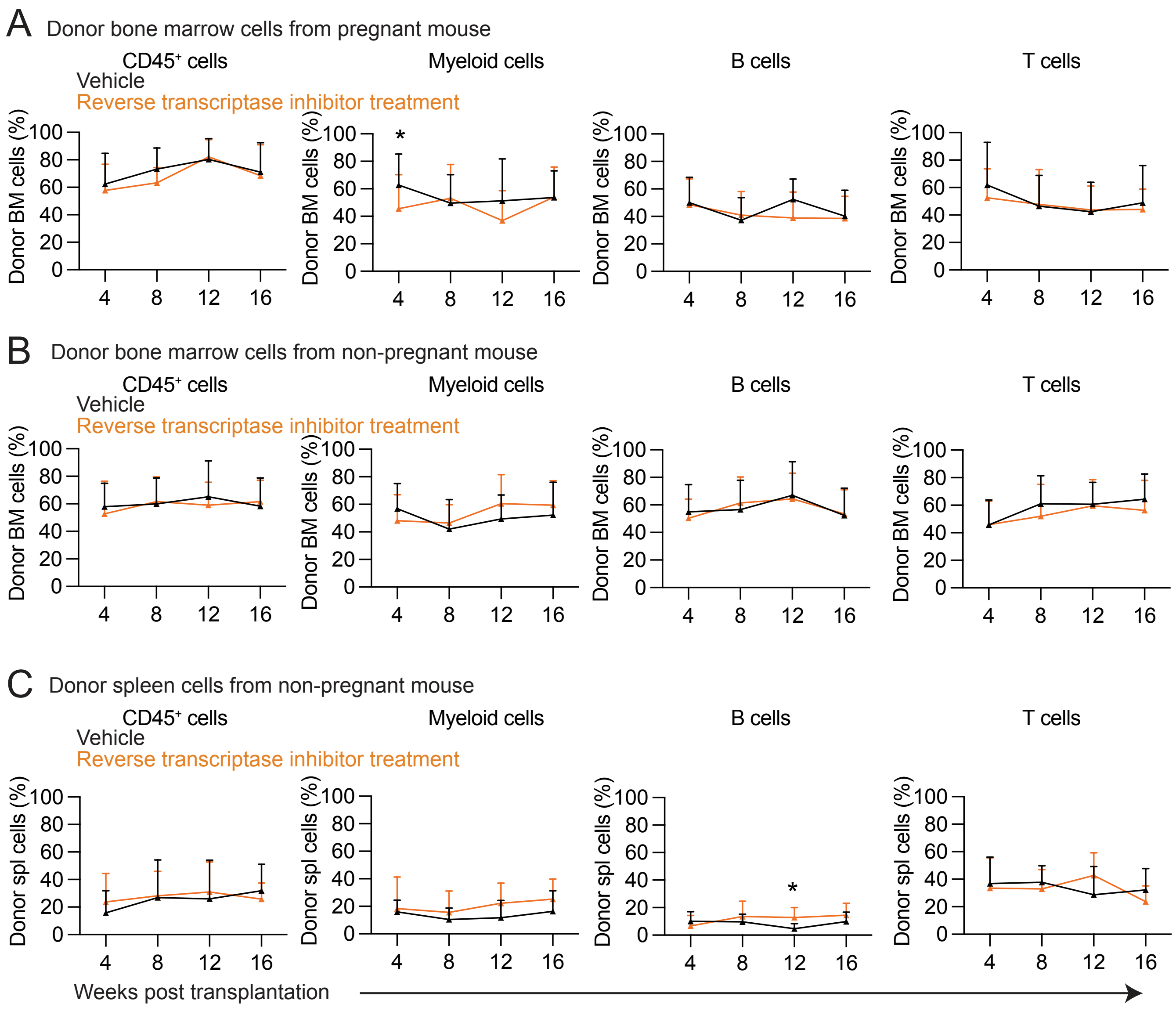

Fig. S5: Treatment with reverse transcriptase inhibitors had no effect on HSC function in non-pregnant female mice. Pregnant and non-pregnant female mice were treated with reverse transcriptase inhibitors or vehicle for 5 days, then unfractionated bone marrow or spleen cells were competitively transplanted into irradiated recipient mice. (**A**) Donor cell reconstitution of CD45$^+$ hematopoietic cells, Mac-1$^+$Gr-1$^+$ myeloid cells, B220$^+$ B cells, and CD3$^+$ T cells in the blood of mice that were competitively transplanted with donor bone marrow cells from pregnant dams treated with reverse transcriptase inhibitors (13 recipients total) or vehicle control (14 recipients total). 5 x 10^5 donor bone marrow cells were transplanted along with 5 x 10^5 competitor bone marrow cells, with 3 donor mice per genotype in 3

independent experiments. Treatment of pregnant mice with reverse transcriptase inhibitors reduced the myeloid reconstituting ability of bone marrow cells but not overall reconstituting ability. (**B**) Donor cell reconstitution in the blood of mice that were competitively transplanted with donor bone marrow cells from non-pregnant mice treated with reverse transcriptase inhibitors (13 recipients total) or vehicle control (12 recipients total). 5 x 10^5 donor bone marrow cells were transplanted along with 5 x 10^5 competitor bone marrow cells, with 3 donor mice per genotype in 3 independent experiments. (**C**) Donor cell reconstitution in the blood of mice that were competitively transplanted with donor spleen cells from non-pregnant mice treated with reverse transcriptase inhibitors or vehicle control (13 recipients total). 1.5 x 10^6 donor spleen cells were transplanted along with 3 x 10^5 competitor bone marrow cells, with 3 donor mice per genotype in 3 independent experiments. All data represent mean ± standard deviation (∗$p < 0.05$; ∗∗$p < 0.01$; ∗∗∗$p < 0.001$). Statistical significance was assessed with nparLD tests followed by Holm-Sidak's multiple comparisons adjustments within cell types of each condition for the overall differences and Mann-Whitney tests for the time points **(A-C)**. All statistical tests were two-sided.

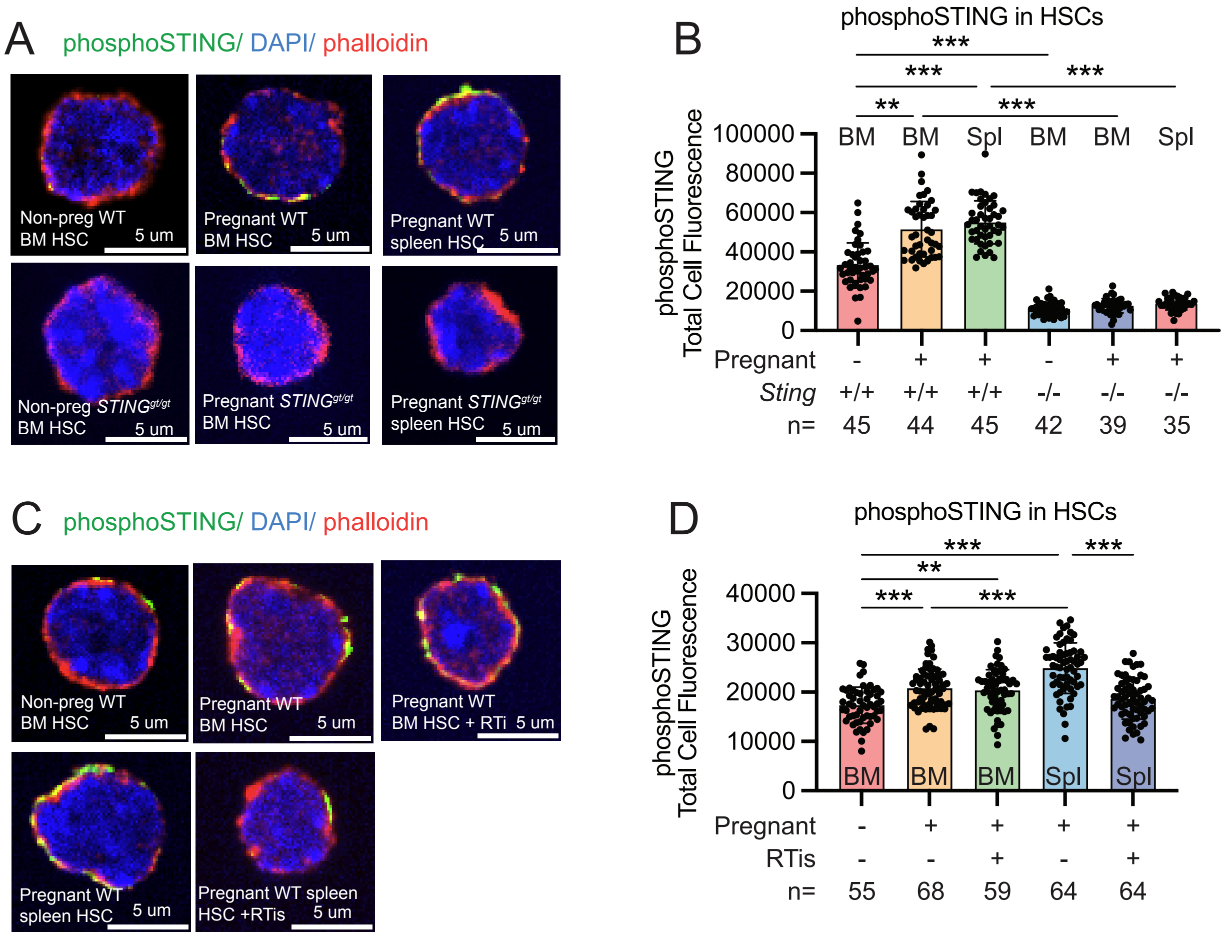

Fig. S6: cGAS-STING activation in HSCs from pregnant mice is attenuated by treatment with reverse transcriptase inhibitors. (**A, B**) We stained HSCs from pregnant or non-pregnant *STING*$^{gt/gt}$ or littermate control mice with anti-phosphoSTING antibody to assess STING activation (*39*) (**A**) and quantitated immunofluorescence staining (**B**). Actin filaments in the cytoplasm were stained with phalloidin (red) and nuclei were stained with DAPI (blue). PhosphoSTING staining increased in HSCs from pregnant as compared to non-pregnant mice but not in STING mutant mice (35 to 45 cells per treatment from 2 mice per treatment). (**C, D**) We stained HSCs from pregnant or non-pregnant mice, that had been treated with reverse transcriptase inhibitors or vehicle control, with anti-phosphoSTING antibody (**C**) and quantitated immunofluorescence staining (**D**). PhosphoSTING staining increased in HSCs from pregnant as compared to non-pregnant mice but treatment with reverse transcriptase inhibitors

reduced phosphoSTING immunofluorescence in HSCs from the spleen (55 to 68 cells per treatment from 1 mouse per treatment). The exact number of cells imaged in each treatment is shown in panels (**B)** and (**D)**. Each dot represents a different cell and all data represent mean ± standard deviation (∗$p < 0.05$; ∗∗$p < 0.01$; ∗∗∗$p < 0.001$). Statistical significance was assessed using a Kruskal-Wallis test followed by Dunn's multiple comparisons adjustments **(B)** or a one-way ANOVA followed by Sidak's multiple comparisons adjustments **(D).** All statistical tests were two-sided.

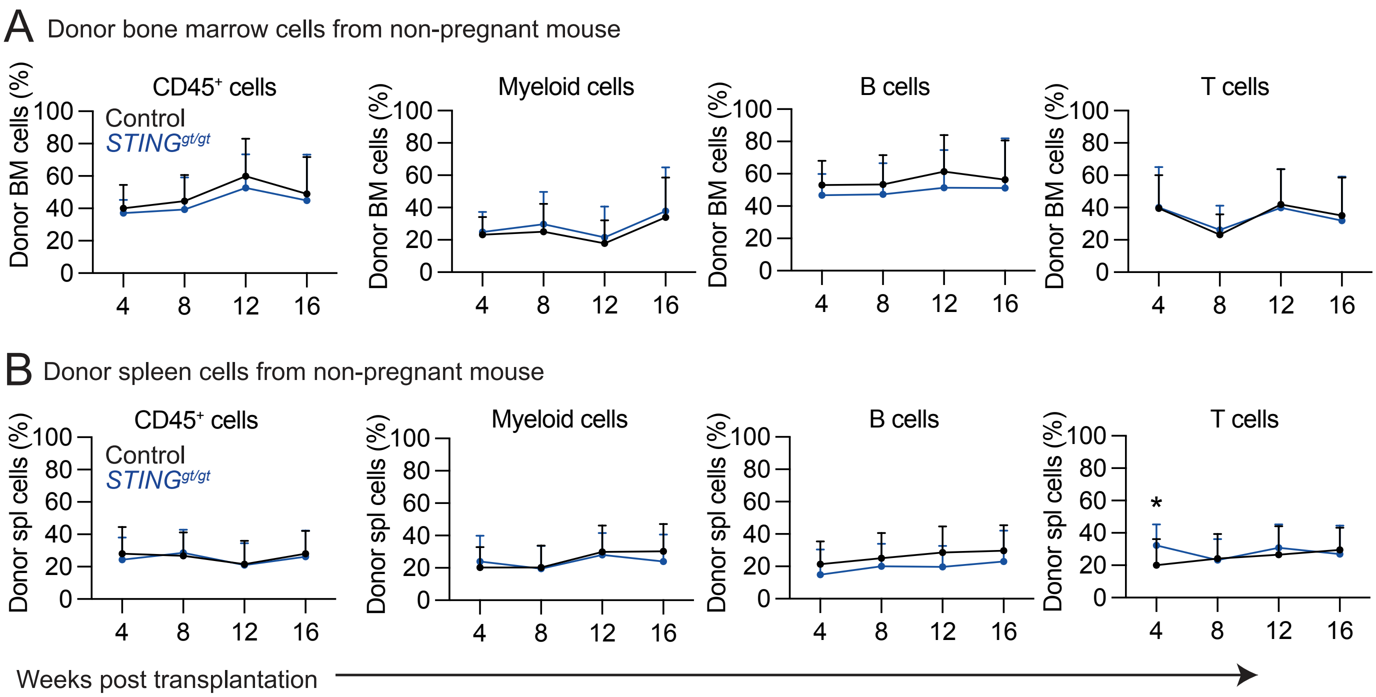

Fig. S7: STING deficiency did not significantly affect the reconstituting potential of bone marrow or spleen cells from non-pregnant female mice. Unfractionated bone marrow or spleen cells from non-pregnant female *STING*$^{gt/gt}$ or littermate control mice were competitively transplanted into irradiated recipient mice. (**A**) Donor cell reconstitution of CD45^{+} hematopoietic cells, Mac-1^{+}Gr-1^{+} myeloid cells, B220^{+} B cells, and CD3^{+} T cells in the blood of mice that were competitively transplanted with donor bone marrow cells from non-pregnant *STING*$^{gt/gt}$ (15 recipients total) or littermate control mice (14 recipients total). 5 x 10^5 donor bone marrow cells from donor were transplanted along with 5 x 10^5 competitor bone marrow cells from competitor, 3 donors per genotype in 3 independent experiments. (**B**) Donor cell reconstitution in the blood of mice that were competitively transplanted with donor spleen cells from non-pregnant *STING*$^{gt/gt}$ or littermate control mice (12 recipients total). 1.5 x 10^6 donor spleen cells from donor, were transplanted along with 3 x 10^5 competitor bone marrow cells from competitor, 3 donors per genotype in 3 independent experiments. All data represent mean ± standard deviation ($*p < 0.05$; $**p < 0.01$; $***p < 0.001$). Statistical significance was assessed with nparLD tests followed by Holm-Sidak's multiple comparisons adjustments within cell types of each condition for the overall differences and Mann-Whitney tests for the time points **(A-C)**. All statistical tests were two-sided.

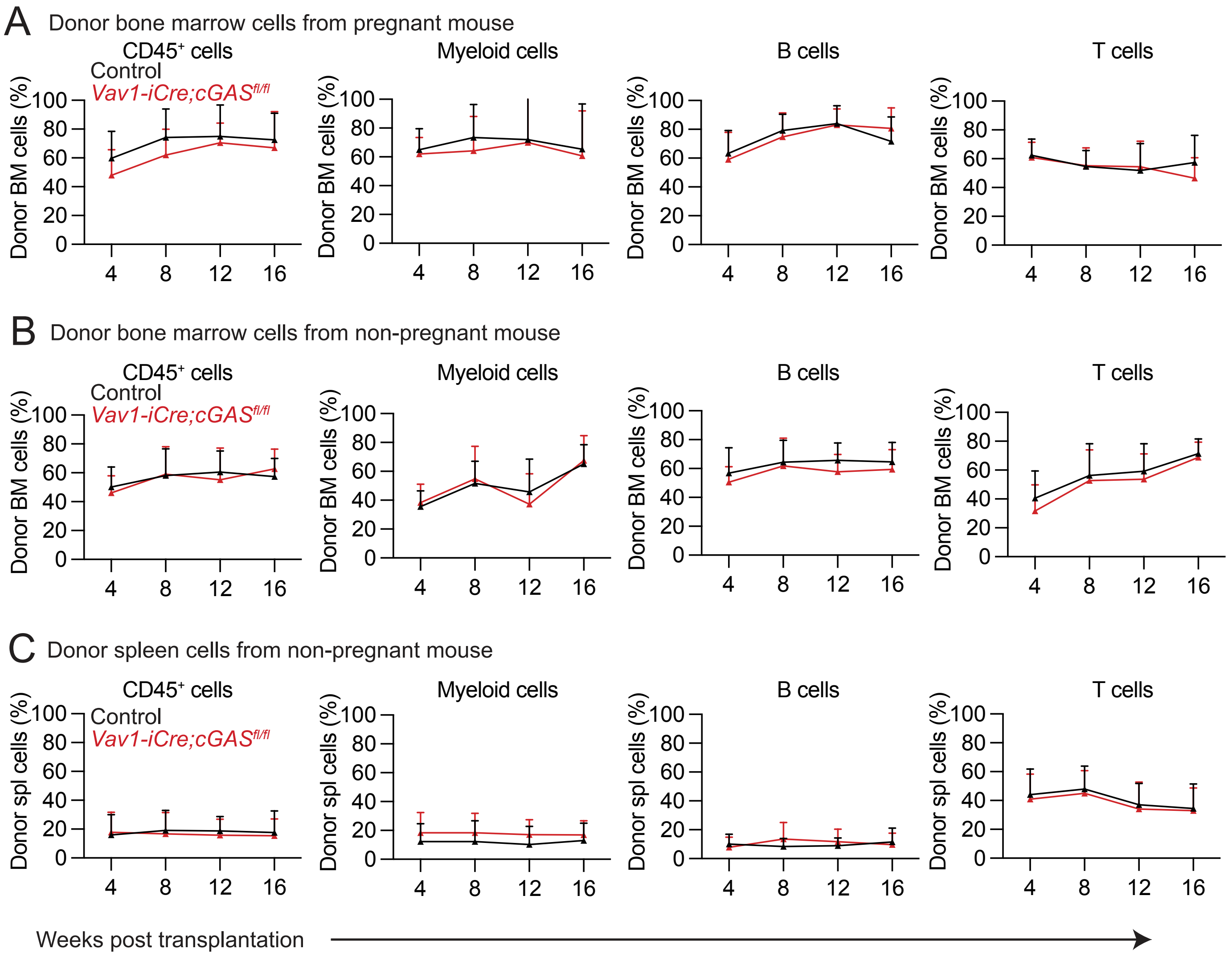

Fig. S8: cGAS deficiency did not significantly affect the reconstituting potential of bone marrow cells from pregnant mice or bone marrow or spleen cells from non-pregnant female mice. (**A**) Unfractionated bone marrow cells from pregnant *Vav-iCre;cGAS*$^{fl/fl}$ or littermate control mice were competitively transplanted into irradiated recipient mice. Donor cell reconstitution of CD45^{+} hematopoietic cells, Mac-1^{+}Gr-1^{+} myeloid cells, B220^{+} B cells, and CD3^{+} T cells in the blood (a total of 11 or 12 (control) recipients. 5 x 10^{5} donor bone marrow cells were transplanted along 5 x 10^{5} competitor bone marrow cells from competitor, 3 donors per genotype in 3 independent experiments. (**B**) Donor cell reconstitution in the blood of recipients of donor bone marrow cells from non-pregnant *Vav1-*

iCre;cGAS$^{fl/fl}$ (15 recipients total) or littermate control mice (14 recipients total). 5 x 10^5 donor bone marrow cells from donor were transplanted along with 5 x 10^5 competitor bone marrow cells, 3 donors per genotype in 3 independent experiments. (**C**) Donor cell reconstitution in the blood of mice that were competitively transplanted with donor spleen cells from non-pregnant *Vav-iCre;cGAS*$^{fl/fl}$ (11 recipients total) or littermate control mice (10 recipients total). 1.5 x 10^6 donor spleen cells from donor were transplanted along with 3 x 10^5 competitor bone marrow cells, 3 donors per genotype in 3 independent experiments. All data represent mean ± standard deviation ($*p < 0.05$; $**p < 0.01$; $***p < 0.001$). Statistical significance was assessed with nparLD tests followed by Holm-Sidak's multiple comparisons adjustments within cell types of each condition for the overall differences and Mann-Whitney tests for the time points **(A-C)**. All statistical tests were two-sided.

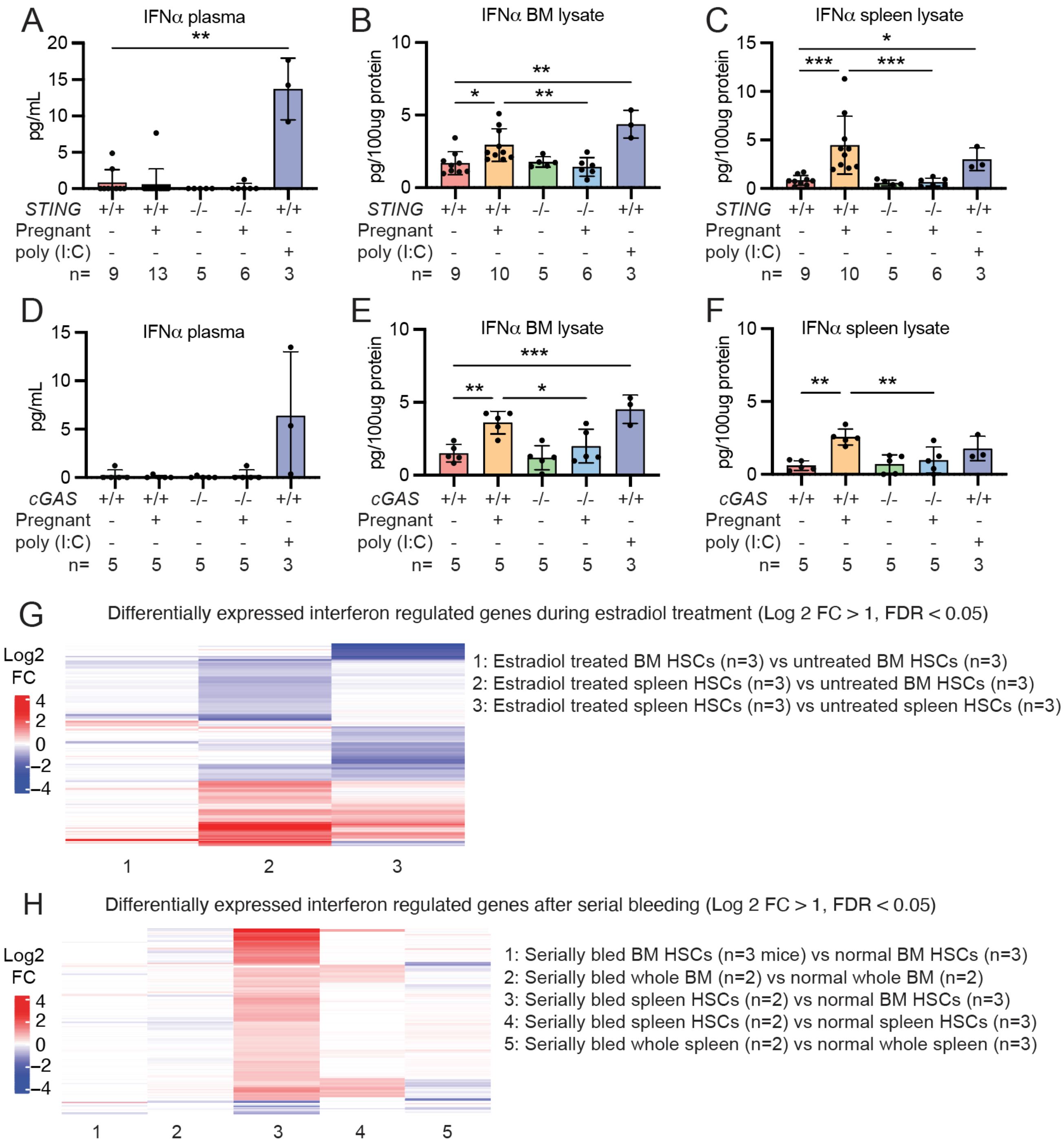

Fig. S9: Interferon alpha production during pregnancy and interferon regulated genes after estradiol treatment or serial bleeding. (**A-C**) ELISA analysis of IFNα in blood plasma (**A**), bone marrow lysate (**B**), or spleen lysate (**C**) from *STING*$^{gt/gt}$ and littermate control mice that were pregnant or

non-pregnant. Some control mice were treated with poly I:C as a positive control for induction of interferon expression (two independent experiments) (*66*). (**D-F**) ELISA analysis of IFNα in blood plasma (**D**), bone marrow lysate (**E**), or spleen lysate (**F**) from *Vav1-iCre;cGAS$^{fl/fl}$* and littermate control mice that were pregnant or non-pregnant (two independent experiments). In panels (**A**) to (**F**) the number of mice per treatment is indicated in each figure panel; each dot represents a different mouse. All data represent mean ± standard deviation (*$p < 0.05$; **$p < 0.01$; ***$p < 0.001$). (**G**) RNA-sequencing of bone marrow and spleen HSCs from estradiol-treated or untreated control mice showed changes in interferon regulated gene expression in spleen HSCs from estradiol-treated mice (log2 fold change > 1, FDR < 0.05). (**H**) RNA-sequencing of bone marrow and spleen HSCs from serially-bled mice and normal control mice showed changes in interferon regulated gene expression in spleen HSCs from serially-bled mice (log2 fold change > 1, FDR < 0.05). Statistical significance was assessed using Kruskal-Wallis tests followed by Dunn's multiple comparisons adjustments (**A, D**) or one-way ANOVAs followed by Sidak's multiple comparisons adjustments (**B, C, E, F**). All statistical tests were two-sided.

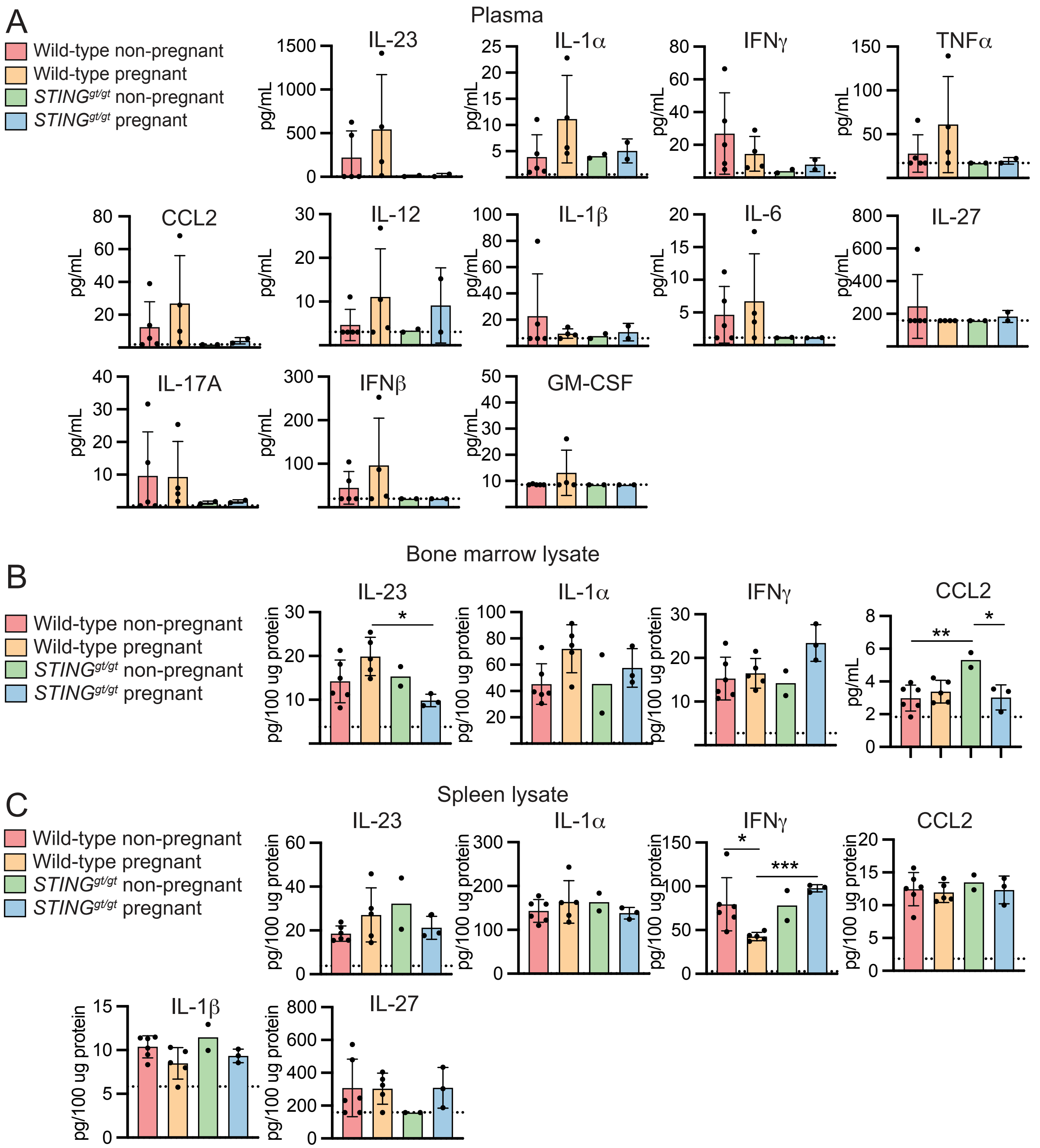

D

Upregulated gene sets in spleen HSCs from pregnant mice as compared to spleen HSCs from non-pregnant mice (NES >1.5, FDR< 0.05)

MSigDB Gene Set Annotation	NES	FDR	Number of Genes Upregulated in Gene Set	Number of Genes in Gene Set
GOBP Type II interferon production	2.04	0.000	39	117
GOBP positive regulation of type II interferon production	1.95	0.002	29	75
GOBP Type II interferon signalling	1.78	0.017	9	29

Fig. S10: Few statistically significant changes in the levels of inflammatory cytokines in blood plasma, bone marrow lysate, or spleen lysate from pregnant as compared to non-pregnant mice. (**A-C**) The levels of inflammatory cytokines were quantitated using Bioplex assays in blood plasma (**A**), bone marrow lysate (**B**), and spleen lysate (**C**), from pregnant or non-pregnant wild-type or *STING*$^{gt/gt}$ mice. Dotted lines show the lower limit of detection for each cytokine. Undetected cytokines are not shown. Each dot represents a different mouse: panels (**A-C**) include 2 to 6 mice per treatment. All data represent mean ± standard deviation (∗$p < 0.05$; ∗∗$p < 0.01$; ∗∗∗$p < 0.001$). **(D)** Gene sets related to type II interferon signaling were enriched in spleen HSCs from pregnant mice as compared to spleen HSCs from non-pregnant mice. In plasma **(A)**, statistical significance was assessed using two-way ANOVAs followed by Sidak's multiple comparisons adjustments for IL-23, IL-1α, and IL-17α, Mann-Whitney tests followed by Holm-Sidak's multiple comparisons adjustments for TNFα, CCL2, IL-12, IL-1β, IL-27, IFNβ, and GM-CSF, or student's *t*-tests followed by Holm-Sidak's multiple comparisons adjustments and Welch's *t*-tests followed by Holm-Sidak's multiple comparisons adjustments for IFNγ and IL-6. In bone marrow lysate **(B)** statistical significance was assessed using two-way ANOVAs followed by Sidak's multiple comparisons adjustments for all cytokines. In spleen lysate **(C)** statistical significance was assessed using *t*-tests with Holm-Sidak's multiple comparisons adjustments for IL-23, two-way ANOVA followed by Sidak's multiple comparisons adjustments for IL-1α, CCL2, and IL-1β, Welch's *t*-tests followed by Holm-Sidak's multiple comparisons adjustments for IFNγ, and Mann-Whitney tests followed by Holm-Sidak's multiple comparisons adjustments for IL-27. All statistical tests were two-sided.

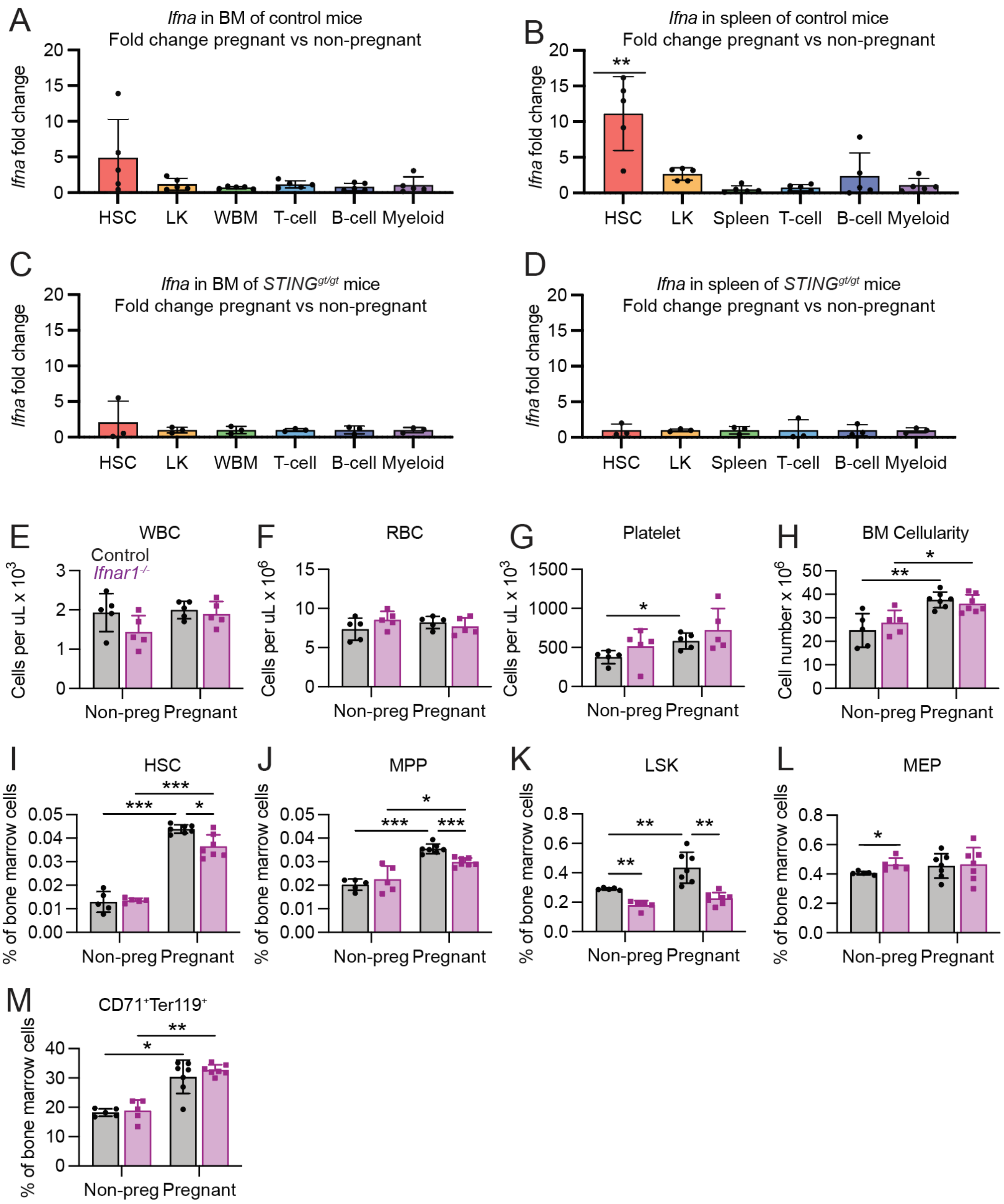
A
Ifna in BM of control mice
Fold change pregnant vs non-pregnant
Ifna fold change
HSC LK WBM T-cell B-cell Myeloid
B
Ifna in spleen of control mice
Fold change pregnant vs non-pregnant
Ifna fold change
HSC LK Spleen T-cell B-cell Myeloid
C
Ifna in BM of STING gt/gt mice
Fold change pregnant vs non-pregnant
D
Ifna in spleen of STING gt/gt mice
Fold change pregnant vs non-pregnant
E
WBC
Control
Ifnar1-/-
Cells per uL x 10^3
Non-preg Pregnant
F
RBC
Cells per uL x 10^6
G
Platelet
Cells per uL x 10^3
H
BM Cellularity
Cell number x 10^6
I
HSC
% of bone marrow cells
J
MPP
K
LSK
L
MEP
M
CD71+Ter119+

Fig. S11: Interferon is induced in HSCs during pregnancy in a STING-dependent manner and *Ifnar1* deficiency reduces stem/progenitor cell frequency in the bone marrow of pregnant mice. (**A-B**) qRT-PCR analysis of *Ifna* transcript levels in sorted bone marrow (**A**) or spleen (**B**) cells from pregnant mice as compared to non-pregnant controls (a total of 5 mice per treatment from 2 independent experiments; each dot represents a different mouse). Data are normalized to transcript levels in non-pregnant controls for each cell population. (**C-D**) qRT-PCR analysis of *Ifna* transcript levels in sorted bone marrow (**C**) or spleen (**D**) cells from pregnant $STING^{gt/gt}$ as compared to non-pregnant $STING^{gt/gt}$ mice (a total of 3 mice per treatment from one experiment). Data are normalized to transcript levels in non-pregnant $STING^{gt/gt}$ mice for each cell population. (**E-M**) We assessed hematopoiesis in pregnant or non-pregnant female $Ifnar1^{-/-}$ or littermate control mice (all panels reflect a total of 5 to 7 mice per treatment from 2 independent experiments): blood cell counts (**E-G**), bone marrow cellularity in one tibia and one femur (**H**), and the frequencies of HSCs (**I**), MPPs (**J**), LSK cells (**K**), MEPs (**L**), and $CD71^+Ter119^+$ erythroid progenitors (**M**) in the bone marrow. The flow cytometry gates are shown in Fig. S1. All data represent mean ± standard deviation (∗$p < 0.05$; ∗∗$p < 0.01$; ∗∗∗$p < 0.001$). Statistical significance was assessed using one-sample *t*-tests **(A-D),** a two-way ANOVA followed by Sidak's multiple comparisons adjustment **(E)**, multiple Student's *t*-tests followed by Holm-Sidak's multiple comparisons adjustments **(F, H)**, Mann-Whitney tests followed by Holm-Sidak's multiple comparisons adjustments **(G, K-M)**, or Welch's *t*-tests followed by Holm-Sidak's multiple comparisons adjustments (**I, J).** All statistical tests were two-sided.

A Flow cytometry gating strategy to obtain Lin^{-}CD34^{+}CD38^{-} cells from human blood

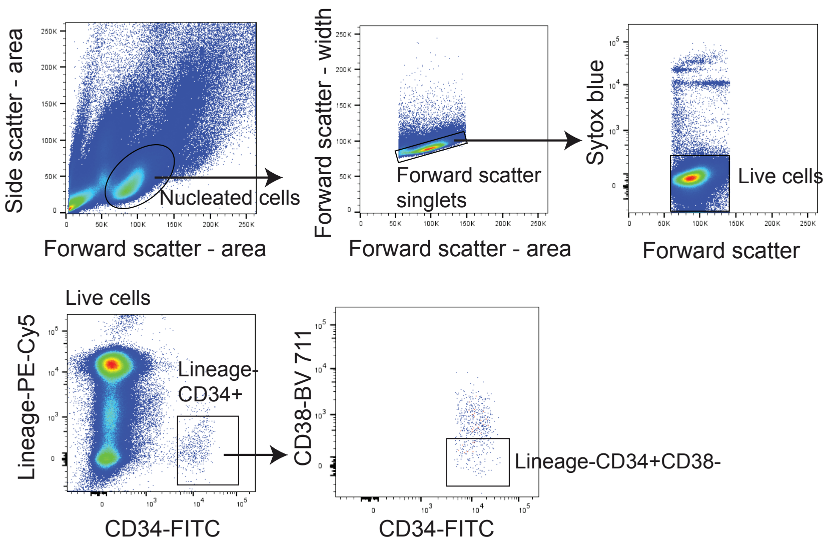

Fig. S12: Flow cytometry gating strategy for the isolation of human HSCs from blood.

Representative flow cytometry gates used to isolate Lineage^{-}CD34^{+}CD38^{-} cells, which are highly enriched for HSCs, from the blood of pregnant and non-pregnant women.

Table S1: Significantly differentially expressed retrotransposons in splenic HSCs from pregnant (E14) mice as compared to bone marrow HSCs from non-pregnant mice (fold change > 2, FDR < 0.05). Fold change reflects splenic HSCs/bone marrow HSCs. Nearly all of these retrotransposons were significantly more highly expressed in splenic HSCs from pregnant mice.

	Fold Change	FDR
IAPEy-int:ERVK:LTR	38.1	1.78E-11
MLT1G1-int:ERVL-MaLR:LTR	32.3	3.11E-03
MER110:ERV1:LTR	25.5	7.74E-03
ERVB5_1-I_MM-int:ERVK:LTR	18.8	9.53E-09
ORR1G-int:ERVL-MaLR:LTR	15.4	1.75E-02
RLTR10D2:ERVK:LTR	14.8	1.25E-05
MER4B-int:ERV1:LTR	12.2	2.31E-02
RLTR1F_Mm:ERVK:LTR	11.7	7.39E-05
RLTR9A3A:ERVK:LTR	11.6	2.91E-09
RLTR13C1:ERVK:LTR	11.5	7.62E-05
IAPLTR4_I:ERVK:LTR	11.3	1.90E-04
MMERVK10D3_LTR:ERVK:LTR	10.1	8.45E-07
RLTR30C_MM:ERV1:LTR	10.1	8.10E-05
RLTR13A1:ERVK:LTR	9.83	3.51E-03
RLTR13D5:ERVK:LTR	9.63	3.59E-05
X9_LINE:L1:LINE	9.46	8.66E-04
Lx2A1:L1:LINE	9.29	1.48E-05
RLTR44D:ERVK:LTR	8.85	8.97E-06
ERVB4_1-I_MM-int:ERVK:LTR	8.74	2.46E-04
RLTR10D:ERVK:LTR	8.56	1.84E-06
LTR48B:ERV1:LTR	8.29	1.10E-05
RLTR44C:ERVK:LTR	7.99	1.71E-05
MURVY-int:ERV1:LTR	7.83	3.72E-03
MLT1G3-int:ERVL-MaLR:LTR	7.82	1.11E-03
ERVB3_1-I_MM-int:ERVK:LTR	7.75	3.80E-03
RMER16A3:ERVK:LTR	7.65	3.53E-05
L1ME5:L1:LINE	7.37	9.53E-05
MuRRS4-int:ERV1:LTR	7.36	3.81E-05
RLTR9F:ERVK:LTR	7.34	2.37E-03
ERVB7_3-LTR_MM:ERVK:LTR	7.06	3.74E-06
MLT1E1A-int:ERVL-MaLR:LTR	7.04	2.64E-02
Lx3A:L1:LINE	6.75	4.07E-03
Lx5b:L1:LINE	6.68	3.93E-05
MLT1J2:ERVL-MaLR:LTR	6.65	1.72E-07

Lx2B:L1:LINE	6.65	3.01E-05
RLTR44B:ERVK:LTR	6.52	2.13E-04
RLTR44A:ERVK:LTR	6.32	1.04E-05
X1_LINE:CR1:LINE	6.27	2.49E-02
RLTR50B:ERVK:LTR	6.26	4.98E-04
Lx4B:L1:LINE	6.23	2.21E-05
ERVB4_1B-I_MM-int:ERVK:LTR	6.22	2.08E-03
LTRIS6:ERV1:LTR	6.22	2.57E-02
MER31-int:ERV1:LTR	6.07	2.70E-02
MER34A1:ERV1:LTR	6.04	2.74E-04
MER57-int:ERV1:LTR	5.98	2.83E-05
Lx4A:L1:LINE	5.94	1.41E-04
LTRIS4A:ERV1:LTR	5.87	1.53E-05
IAP-d-int:ERVK:LTR	5.87	3.35E-04
Lx2A:L1:LINE	5.75	9.95E-04
MuLV-int:ERV1:LTR	5.66	3.08E-02
Lx3C:L1:LINE	5.64	7.68E-03
BGLII:ERVK:LTR	5.60	1.00E-02
MLTR31A_MM:ERVK:LTR	5.57	1.40E-04
RLTR13B1:ERVK:LTR	5.55	6.35E-03
RLTR20B3:ERVK:LTR	5.52	1.24E-04
RMER16B2:ERVK:LTR	5.48	1.12E-02
Lx2B2:L1:LINE	5.46	7.34E-03
RLTR10F:ERVK:LTR	5.41	2.42E-02
RLTR31M:ERVK:LTR	5.37	9.92E-04
Lx2:L1:LINE	5.36	5.09E-04
Lx5:L1:LINE	5.23	2.05E-04
MLTR31E_MM:ERVK:LTR	5.15	4.12E-04
RMER13B:ERVK:LTR	5.08	1.39E-05
ETnERV2-int:ERVK:LTR	5.03	2.19E-03
RLTR20A:ERVK:LTR	5.02	1.26E-03
RLTR45:ERVK:LTR	4.99	3.02E-03
BGLII_Mur:ERVK:LTR	4.95	1.09E-03
RLTR6-int:ERV1:LTR	4.93	2.69E-04
RLTR31B_Mm:ERVK:LTR	4.91	9.99E-04
RLTR9E:ERVK:LTR	4.80	9.89E-04
LTR33C:ERVL:LTR	4.64	3.30E-02
ERVB3_1-LTR_MM:ERVK:LTR	4.54	7.48E-03
RLTR20A2B_MM:ERVK:LTR	4.51	6.18E-04

IAPLTR2b:ERVK:LTR	4.50	1.33E-03
MLTR18_MM:ERVK:LTR	4.49	3.00E-06
MER77B:ERVL:LTR	4.47	2.18E-04
IAPEY2_LTR:ERVK:LTR	4.42	7.78E-03
HERV16-int:ERVL:LTR	4.37	1.59E-02
IAPLTR2a2_Mm:ERVK:LTR	4.36	1.34E-07
MLT1H1:ERVL-MaLR:LTR	4.35	5.80E-03
RLTR34C_MM:ERVK:LTR	4.34	3.82E-05
Lx5c:L1:LINE	4.34	1.28E-03
IAPLTR4:ERVK:LTR	4.31	1.53E-05
IAP1-MM_LTR:ERVK:LTR	4.25	4.99E-03
RLTR20D:ERVK:LTR	4.13	2.46E-04
MER31A:ERV1:LTR	4.08	5.13E-04
L1_Mus4:L1:LINE	4.05	9.95E-04
Lx3_Mus:L1:LINE	4.04	2.21E-02
Lx6:L1:LINE	4.03	1.29E-03
L1Md_F:L1:LINE	4.02	1.28E-03
ERV3-16A3_I-int:ERVL:LTR	3.97	9.35E-03
RLTR12D:ERVK:LTR	3.88	4.40E-03
IAPLTR2a:ERVK:LTR	3.81	1.67E-03
RLTR41C:ERV1:LTR	3.79	1.10E-02
RLTR13C2:ERVK:LTR	3.76	2.40E-03
RLTR26_Mus:ERVK:LTR	3.76	1.28E-03
BGLII_Mus:ERVK:LTR	3.74	1.90E-02
LTR84b:ERVL:LTR	3.73	4.62E-03
RLTR10B2:ERVK:LTR	3.72	6.97E-03
MuRRS-int:ERV1:LTR	3.72	1.67E-03
ERVB4_2-I_MM-int:ERVK:LTR	3.72	1.00E-02
RMER19C:ERVK:LTR	3.71	1.46E-04
IAPEY_LTR:ERVK:LTR	3.70	2.61E-03
IAPLTR1a_Mm:ERVK:LTR	3.69	1.32E-06
RMER16:ERVK:LTR	3.67	4.43E-03
MMERVK9E_I-int:ERVK:LTR	3.64	4.73E-03
Lx:L1:LINE	3.62	2.51E-03
LTR16A1:ERVL:LTR	3.59	9.46E-03
MLT2B3:ERVL:LTR	3.57	2.71E-03
MLT1E3:ERVL-MaLR:LTR	3.56	5.27E-05
RLTR13E:ERVK:LTR	3.53	1.40E-04
RLTR13C3:ERVK:LTR	3.51	2.98E-03

RLTR16C_MM:ERVK:LTR	3.49	5.15E-03
RLTR13D3A:ERVK:LTR	3.48	4.06E-02
ERVB4_3-I_MM-int:ERVK:LTR	3.47	2.16E-02
RLTR6C_Mm:ERV1:LTR	3.45	2.00E-02
MLT1A1-int:ERVL-MaLR:LTR	3.44	1.29E-02
RodERV21-int:ERV1:LTR	3.44	4.95E-03
MLT1F1:ERVL-MaLR:LTR	3.43	2.00E-04
L1Md_A:L1:LINE	3.43	6.45E-05
L1Md_Gf:L1:LINE	3.43	1.60E-03
RLTR34D_MM:ERVK:LTR	3.41	5.36E-03
MLTR31C_MM:ERVK:LTR	3.40	3.05E-02
L1_Mur2:L1:LINE	3.40	2.32E-03
RLTR17:ERVK:LTR	3.40	5.67E-03
MLT1J1:ERVL-MaLR:LTR	3.38	1.17E-02
RLTR31A_Mm:ERVK:LTR	3.36	1.29E-02
MLT1K:ERVL-MaLR:LTR	3.36	2.15E-03
MLT1H:ERVL-MaLR:LTR	3.32	3.12E-03
RLTR41:ERV1:LTR	3.32	6.29E-03
Lx10:L1:LINE	3.29	3.61E-03
MLT1E:ERVL-MaLR:LTR	3.27	4.59E-03
ERVB7_2B-LTR_MM:ERVK:LTR	3.26	1.72E-02
RLTR9A:ERVK:LTR	3.21	2.06E-02
BGLII_B:ERVK:LTR	3.19	4.76E-03
RLTR16:ERVK:LTR	3.18	1.11E-02
RLTR10:ERVK:LTR	3.15	8.77E-03
MLT1H-int:ERVL-MaLR:LTR	3.14	1.20E-02
MLT1I:ERVL-MaLR:LTR	3.13	1.08E-04
RMER20B:ERVK:LTR	3.11	6.51E-03
Lx8b:L1:LINE	3.09	4.12E-03
RLTR49:ERVK:LTR	3.07	8.28E-03
RLTR10-int:ERVK:LTR	3.06	7.01E-03
MLT1H2:ERVL-MaLR:LTR	3.05	1.30E-02
MLT2B1:ERVL:LTR	3.05	1.11E-02
RMER6C:ERVK:LTR	3.04	8.72E-03
ERVB4_1B-LTR_MM:ERVK:LTR	3.04	1.40E-02
L1_Mus2:L1:LINE	3.02	3.01E-03
RMER13A2:ERVK:LTR	3.01	5.09E-03
LTRIS_Mm:ERV1:LTR	3.01	1.35E-02
Lx8:L1:LINE	3.00	5.61E-03

RMER3D-int:ERVK:LTR	3.00	4.44E-03
MER92B:ERV1:LTR	2.99	1.39E-03
RLTR10A:ERVK:LTR	2.98	1.35E-02
Lx7:L1:LINE	2.97	4.73E-03
RMER5:ERV1:LTR	2.97	6.18E-04
L1MCc:L1:LINE	2.97	3.02E-02
RLTR11B:ERVK:LTR	2.96	1.16E-02
RLTR20B2:ERVK:LTR	2.95	1.08E-02
ERVB4_1C-LTR_Mm:ERVK:LTR	2.94	2.21E-02
RLTR12A:ERVK:LTR	2.94	1.29E-02
RLTR11C_MM:ERVK:LTR	2.94	2.42E-03
LTR33:ERVL:LTR	2.92	7.39E-04
ERVL-E-int:ERVL:LTR	2.92	4.32E-03
RLTR26:ERVK:LTR	2.90	3.38E-02
RLTR16B_MM:ERVK:LTR	2.90	2.66E-02
RLTR20B5_MM:ERVK:LTR	2.90	2.61E-02
MER54B:ERVL:LTR	2.89	4.76E-02
MLTR73:ERV1:LTR	2.89	7.44E-03
L1_Mur1:L1:LINE	2.88	9.46E-03
IAPEY3-int:ERVK:LTR	2.87	1.81E-02
RMER17B:ERVK:LTR	2.87	7.60E-03
MMERVK9C_I-int:ERVK:LTR	2.86	3.82E-02
MLTR31F_MM:ERVK:LTR	2.85	2.33E-02
RLTR13A3:ERVK:LTR	2.81	2.77E-02
BGLII_B2:ERVK:LTR	2.81	9.30E-03
RMER19A:ERVK:LTR	2.81	1.33E-02
ORR1B2-int:ERVL-MaLR:LTR	2.79	1.26E-02
RLTR10C:ERVK:LTR	2.76	8.34E-03
RLTR20B3A_MM:ERVK:LTR	2.76	3.48E-02
ETnERV-int:ERVK:LTR	2.74	3.27E-02
MLT1C:ERVL-MaLR:LTR	2.71	2.97E-03
RLTR13D6:ERVK:LTR	2.71	3.17E-02
RLTR22_Mus:ERVK:LTR	2.70	7.16E-04
RMER16-int:ERVK:LTR	2.70	3.93E-03
MER74B:ERVL:LTR	2.69	9.37E-04
IAPLTR3-int:ERVK:LTR	2.68	9.15E-03
RLTR12BD_Mm:ERVK:LTR	2.67	3.15E-02
LTR16B2:ERVL:LTR	2.66	3.19E-02
RMER20C_Mm:ERVK:LTR	2.66	2.28E-02

ORR1C1:ERVL-MaLR:LTR	2.64	8.71E-03
L1_Mus1:L1:LINE	2.64	9.50E-03
MLT1G1:ERVL-MaLR:LTR	2.63	9.79E-04
L1_Mus3:L1:LINE	2.63	9.32E-03
RLTR6B_Mm:ERV1:LTR	2.63	2.47E-02
RLTR12G:ERVK:LTR	2.62	3.49E-02
MLT1A0:ERVL-MaLR:LTR	2.62	3.09E-03
RLTR11A2:ERVK:LTR	2.61	1.59E-02
RLTR13D1:ERVK:LTR	2.61	5.29E-03
RMER19B2:ERVK:LTR	2.60	1.58E-02
RMER15:ERVL:LTR	2.60	4.10E-03
RLTR44-int:ERVK:LTR	2.59	2.93E-02
LTRIS_Mus:ERV1:LTR	2.59	1.67E-02
RLTR1D2_MM:ERV1:LTR	2.57	3.50E-02
X7B_LINE:CR1:LINE	2.56	2.24E-02
RLTR12E:ERVK:LTR	2.56	9.02E-03
ORR1B2:ERVL-MaLR:LTR	2.56	4.79E-03
LTR16C:ERVL:LTR	2.55	9.04E-03
RLTR35B_MM:ERVL:LTR	2.55	6.11E-03
RMER1B:RMER1B:Other	2.54	1.43E-02
RLTR14:ERV1:LTR	2.52	1.74E-02
MLT1E2:ERVL-MaLR:LTR	2.52	3.93E-03
RLTR9D:ERVK:LTR	2.51	2.64E-02
RLTR23:ERV1:LTR	2.51	2.92E-02
LTRIS2:ERV1:LTR	2.50	2.61E-02
MT2A:ERVL:LTR	2.49	1.37E-02
MTE2b-int:ERVL-MaLR:LTR	2.49	1.40E-02
ORR1A4:ERVL-MaLR:LTR	2.48	1.45E-02
MLT1J:ERVL-MaLR:LTR	2.48	1.03E-02
RLTR20B4_MM:ERVK:LTR	2.48	2.64E-02
MTEb-int:ERVL-MaLR:LTR	2.47	2.50E-03
LTR40a:ERVL:LTR	2.47	2.29E-02
ORR1B1:ERVL-MaLR:LTR	2.46	5.15E-03
Lx9:L1:LINE	2.45	1.39E-02
L1_Mm:L1:LINE	2.45	5.37E-03
L1VL1:L1:LINE	2.45	1.49E-02
RLTR20A3_MM:ERVK:LTR	2.44	2.42E-02
RMER17A2:ERVK:LTR	2.43	1.07E-02
L1VL4:L1:LINE	2.42	1.20E-02

ORR1F:ERVL-MaLR:LTR	2.41	8.82E-03
MLT1F-int:ERVL-MaLR:LTR	2.41	2.67E-02
MERV1_LTR:ERV1:LTR	2.39	4.78E-02
MLT1A1:ERVL-MaLR:LTR	2.38	1.27E-02
RMER6D:ERVK:LTR	2.37	2.31E-02
MER89:ERV1:LTR	2.36	3.06E-02
MLTR11B:ERVK:LTR	2.35	2.53E-02
MLT1L:ERVL-MaLR:LTR	2.35	8.71E-03
RLTR34B_MM:ERVK:LTR	2.35	1.23E-02
MLTR14:ERV1:LTR	2.34	2.33E-02
RLTR17D_Mm:ERVK:LTR	2.33	1.80E-03
MLT1F:ERVL-MaLR:LTR	2.32	2.72E-02
MLT1B:ERVL-MaLR:LTR	2.32	1.59E-02
RLTR12B:ERVK:LTR	2.31	2.88E-02
RLTR11A:ERVK:LTR	2.27	3.13E-02
RLTR31B2:ERVK:LTR	2.27	1.49E-02
RLTR4_MM-int:ERV1:LTR	2.27	3.08E-02
LTR78:ERV1:LTR	2.27	4.54E-02
MMETn-int:ERVK:LTR	2.25	1.48E-02
LTR33B:ERVL:LTR	2.25	2.79E-02
IAP1-MM_I-int:ERVK:LTR	2.24	2.29E-02
RLTR13G:ERVK:LTR	2.24	1.03E-02
MLT1E1:ERVL-MaLR:LTR	2.21	2.93E-02
MYSERV16_I-int:ERVK:LTR	2.17	3.05E-02
ORR1F-int:ERVL-MaLR:LTR	2.14	2.28E-02
LTR33A_:ERVL:LTR	2.14	4.77E-02
RLTR53_Mm:ERVK:LTR	2.14	7.52E-03
MMVL30-int:ERV1:LTR	2.14	2.74E-04
ORR1D2-int:ERVL-MaLR:LTR	2.12	2.54E-02
L1M6:L1:LINE	2.11	3.59E-02
MT-int:ERVL-MaLR:LTR	2.11	3.66E-02
Lx3B:L1:LINE	2.10	1.63E-02
IAPA_MM-int:ERVK:LTR	2.09	2.35E-02
ERVL-B4-int:ERVL:LTR	2.06	2.98E-02
L1MCb:L1:LINE	2.05	3.37E-02
MLT1D:ERVL-MaLR:LTR	2.04	2.90E-02
RMER15-int:ERVL:LTR	2.04	3.32E-02
L1M7:L1:LINE	2.02	5.54E-03
MTB_Mm:ERVL-MaLR:LTR	2.01	3.68E-02

RLTR1A2_MM:ERV1:LTR	2.01	2.31E-04
RLTR48B:ERV1:LTR	0.486	5.36E-03
ORR1D-int:ERVL-MaLR:LTR	0.436	1.57E-03
LTR68:ERV1:LTR	0.291	9.08E-03
X7C_LINE:CR1:LINE	0.128	1.86E-03

Table S2. Cell populations analyzed by flow cytometry in this study

Cell population	Abbreviation	Markers	Reference
**Mouse**			
Hematopoietic stem cells	HSC	$CD150^{+}CD48^{-}Lin^{-}Sca1^{+}c\text{-}kit^{+}$	(*67*)
Multipotent progenitors	MPP	$CD150^{-}CD48^{-}Lin^{-}Sca1^{+}c\text{-}kit^{+}$	(*67*)
Hematopoietic stem and progenitor cells	LSK	$Lin^{-}Sca1^{+}c\text{-}kit^{+}$	(*68*)
Megakaryocyte-erythrocyte progenitors	MEP	$Lin^{-}Sca1^{-}c\text{-}kit^{+}CD34^{-}$ $CD16/32^{-}$	(*69*)
Myeloid progenitors	LK	$Lin^{-}Sca1^{-}c\text{-}kit^{+}$	
T cells		$CD3^{+}$	
B cells		$B220^{+}$	
Myeloid cells		$Mac\text{-}1^{+}Gr\text{-}1^{+}$	
Erythroid progenitors		$CD71^{+}Ter119^{+}$	
**Human**			
Hematopoietic stem cells	HSC	$Lin^{-}CD34^{+}CD38^{-}$	(*49*)

Table S3. Antibodies used in this study.

Reagent or Resource	Source	Identifier	Catalog No.	Dilution
**Antibodies**				
α-mouse Gr1 FITC	Tonbo Biosciences	Clone RB6-8C5; RRID:AB_2621721	35-5931	1:200
α-mouse Gr1 APC	Tonbo Biosciences	Clone RB6-8C5; RRID:AB_2621610	20-5931	1:200
α-mouse Gr1 PE	Tonbo Biosciences	Clone RB6-8C5; RRID:AB_2621803	50-5931	1:200
α-mouse CD2 FITC	Tonbo Biosciences	Clone RM2-5; RRID:AB_2621657	35-0021	1:200
α-mouse CD2 PE	Tonbo Biosciences	Clone RM2-5; RRID:AB_2621728	50-0021	1:200
α-mouse CD2 APC	Biolegend	Clone RM2-5; RRID:AB_2563090	100112	1:200
α-mouse CD5 FITC	Biolegend	Clone 53-7.3; RRID:AB_312734	100606	1:200
α-mouse CD5 PE	Biolegend	Clone 53-7.3; RRID:AB_312736	100608	1:200
α-mouse CD5 APC	Biolegend	Clone 53-7.3; RRID:AB_2563929	100626	1:200
α-mouse CD3 FITC	Biolegend	Clone 17A2; RRID:AB_312661	100204	1:200
α-mouse CD3 PE	Tonbo Biosciences	Clone 17A2; RRID:AB_2621731	50-0032	1:200
α-mouse CD3 APC	Tonbo Biosciences	Clone 17A2; RRID:AB_2621538	20-0032	1:200
α-mouse CD8a FITC	Tonbo Biosciences	Clone 53-6.7; RRID:AB_2621671	35-0081	1:200
α-mouse CD8a PE	Tonbo Biosciences	Clone 53-6.7; RRID:AB_2621741	50-0081	1:200
α-mouse CD8a APC	Biolegend	Clone 53-6.7; RRID:AB_312751	100711	1:200
α-human/mouse B220 FITC	Tonbo Biosciences	Clone RA3-6B2; RRID:AB_2621690	35-0452	1:200
α-human/mouse B220 APC	Tonbo Biosciences	Clone RA3-6B2; RRID:AB_2621574	20-0452	1:200
α-human/mouse B220 Percp-Cy5.5	Tonbo Biosciences	Clone RA3-6B2; RRID:AB_2621892	65-0452	1:200
α-human/mouse B220 PE	Tonbo Biosciences	Clone RA3-6B2; RRID:AB_2621764	50-0452	1:200
α-mouse CD45.1 FITC	Biolegend	Clone A20; RRID:AB_313494	110705	1:200
α-mouse CD45.1 PE-Cy7	Tonbo Biosciences	Clone A20; RRID:AB_2621850	60-0453	1:200
α-mouse CD45.2 VioletFluor 450	Tonbo Biosciences	Clone 104; RRID:AB_2621950	75-0454	1:200
α-mouse Ter119 FITC	Tonbo Biosciences	Clone TER-119; RRID:AB_2621720	35-5921	1:200
α-mouse Ter119 PE	Tonbo Biosciences	Clone TER-119; RRID:AB_2621802	50-5921	1:200
α-mouse Ter119 APC	Tonbo Biosciences	Clone TER-119; RRID:AB_2621609	20-5921	1:200

α-mouse CD71 FITC	eBioscience	Clone R17217; RRID:AB_465124	11-0711-82	1:200
α-mouse c-kit APC-eFluor780	eBioscience	Clone 2B8; RRID:AB_1272213	47-1171-80	1:200
α-mouse Sca1 Percp-Cy5.5	Thermo Fisher Scientific	Clone D7; RRID:AB_914370	45-5981-80	1:200
α-mouse CD150 PE	Biolegend	Clone TC15-12F12.2; RRID:AB_313682	115903	1:200
α-mouse CD150 PE-Cy7	Biolegend	Clone TC15-12F12.2; RRID:AB_439796	115913	1:200
α-mouse CD150 APC	Biolegend	Clone TC15-12F12.2; RRID:AB_493461	115909	1:200
α-mouse CD48 APC	Biolegend	Clone HM48-1; RRID:AB_571996	103411	1:200
α-mouse CD48 PE-Cy7	Biolegend	Clone HM48-1; RRID:AB_2075049	103424	1:200
α-mouse CD48 Percp-Cy5.5	Biolegend	Clone HM48-1; RRID:AB_2075051	103422	1:200
α-mouse Mac-1 (CD11b) APC-efluor780	Thermo Fisher Scientific	Clone M1/70; RRID:AB_1603193	47-0112-82	1:200
α-mouse CD16/32 AF700	eBioscience	Clone 93; RRID:AB_493994	56-0161-82	1:200
α-mouse CD45 FITC	Tonbo Biosciences	Clone 30-F11; RRID:AB_2621689	35-0451	1:200
α-mouse CD45 APC	Tonbo Biosciences	Clone 30-F11; RRID:AB_ 2621573	20-0451	1:200
α-mouse CD45 PE	Biolegend	Clone 30-F11; RRID: AB_312971	103106	1:200
α-mouse phosphoSTING	Thermo Fisher Scientific	Polyclonal; RRID: AB_2817102	PA5-105674	1:500
α-rabbit IgG Alexa Fluor 647	Thermo Fisher Scientific	Polyclonal; RRID: AB_2633282	A32733	1:1000
α-human CD3 PECy5	Biolegend	Clone: HIT3a, RRID: AB_315056	406506	1:200
α-human CD4 PECy5	Biolegend	Clone: RPA-T4, RRID: AB_314077	300509	1:200
α-human CD8 PECy5	Biolegend	Clone: RPA-T8, RRID: AB_2904367	344769	1:200
α-human CD14 PECy5	Biolegend	Clone: M5E2, RRID: AB_2860766	301863	1:200
α-human CD235 PECy5	Biolegend	Clone: HIR2, RRID: AB_314623	306605	1:200
α-human CD56 PECy5	Biolegend	Clone: MEM-188, RRID: AB_314449	304607	1:200
α-human CD34 PECy5	Biolegend	Clone: 581, RRID: AB_830758	119311	1:200
α-human CD38 PECy5	Biolegend	Clone: HIT2, RRID: AB_314359	303507	1:200

Table S4. Primers used in this study

Primer	Application	Primer sequence 5'-3'
STING F	Genotyping	GAT CCG AAT GTT CAA TCA GC
STING R	Genotyping	CGA TTC TTG ATG CCA GCA C
Vav1-iCre F	Genotyping	AGATGCCAGGACATCAGGAACC
Vav1-iCre R	Genotyping	ATCAGCCACACCAGACACAGAG
cGAS Floxed F	Genotyping	CCA AAG AAG CAG TCT AAG ACT AGA GT
cGAS Floxed R	Genotyping	TTT TAA GAC CGG GTC TTG CTT TGT AGA
Ifnar F Common	Genotyping	ACT CAG GTT CGC TCC ATC AG
Ifnar R WT	Genotyping	CTT TTA ACC ACT TCG CCT CGT
Ifnar R Mutant	Genotyping	GAA CCT GAG GCT GTC GAA GG
Actb F	qPCR	CAC TGT CGA GTC GCG TCC
Actb R	qPCR	TCA TCC ATG GCG AAC TGG TG
Ifna F	qPCR	ACT CAT AAC CTC AGG AAC AAG
Ifna R	qPCR	CTT TGA TGT GAA GAT GTT CAG
